# Prematurity, ventricular septal defect and dysmorphisms are independent predictors of pathogenic copy number variants: a retrospective study on array-CGH results and phenotypical features of 293 children with neurodevelopmental disorders and/or multiple congenital anomalies

**DOI:** 10.1186/s13052-018-0467-z

**Published:** 2018-03-09

**Authors:** I. Maini, I. Ivanovski, O. Djuric, S. G. Caraffi, E. Errichiello, M. Marinelli, F. Franchi, V. Bizzarri, S. Rosato, M. Pollazzon, C. Gelmini, M. Malacarne, C. Fusco, G. Gargano, S. Bernasconi, O. Zuffardi, L. Garavelli

**Affiliations:** 1 Clinical Genetics Unit, Maternal and Child Health Department, AUSL-IRCCS of Reggio Emilia, Reggio Emilia, Italy; 2 Child Neuropsychiatry Unit, Maternal and Child Health Department, AUSL-IRCCS of Reggio Emilia, Reggio Emilia, Italy; 3 Department of Surgical, Medical, Dental and Morphological Sciences with Interest in Transplant, Oncology and Regenerative Medicine, University of Modena and Reggio Emilia, Modena, Italy; 4 Institute of Epidemiology, School of Medicine, University of Belgrade, Belgrade, Serbia; 5 Department of Molecular Medicine, University of Pavia, Pavia, Italy; 6 Laboratory of Genetics, Maternal and Child Health Department, AUSL-IRCCS of Reggio Emilia, Reggio Emilia, Italy; 7 Division of Medical Genetics, Galliera Hospital, Genoa, Italy; 8 Neonatal Intensive Care Unit (NICU), Maternal and Child Health Department, AUSL-IRCCS of Reggio Emilia, Reggio Emilia, Italy; 9 Former Director Pediatric Department, University of Parma, Parma, Italy; 10 Santa Maria Nuova Hospital, viale Risorgimento 80, 42123 Reggio Emilia, Italy

**Keywords:** Array-CGH, Neurodevelopmental disorders, Multiple congenital anomalies, Dysmorphisms, Interpretation

## Abstract

**Background:**

Since 2010, array-CGH (aCGH) has been the first-tier test in the diagnostic approach of children with neurodevelopmental disorders (NDD) or multiple congenital anomalies (MCA) of unknown origin. Its broad application led to the detection of numerous variants of uncertain clinical significance (VOUS). How to appropriately interpret aCGH results represents a challenge for the clinician.

**Method:**

We present a retrospective study on 293 patients with age range 1 month - 29 years (median 7 years) with NDD and/or MCA and/or dysmorphisms, investigated through aCGH between 2005 and 2016. The aim of the study was to analyze clinical and molecular cytogenetic data in order to identify what elements could be useful to interpret unknown or poorly described aberrations. Comparison of phenotype and cytogenetic characteristics through univariate analysis and multivariate logistic regression was performed.

**Results:**

Copy number variations (CNVs) with a frequency < 1% were detected in 225 patients of the total sample, while 68 patients presented only variants with higher frequency (heterozygous deletions or amplification) and were considered to have negative aCGH. Proved pathogenic CNVs were detected in 70 patients (20.6%). Delayed psychomotor development, intellectual disability, intrauterine growth retardation (IUGR), prematurity, congenital heart disease, cerebral malformations and dysmorphisms correlated to reported pathogenic CNVs. Prematurity, ventricular septal defect and dysmorphisms remained significant predictors of pathogenic CNVs in the multivariate logistic model whereas abnormal EEG and limb dysmorphisms were mainly detected in the group with likely pathogenic VOUS.

A flow-chart regarding the care for patients with NDD and/or MCA and/or dysmorphisms and the interpretation of aCGH has been made on the basis of the data inferred from this study and literature.

**Conclusion:**

Our work contributes to make the investigative process of CNVs more informative and suggests possible directions in aCGH interpretation and phenotype correlation.

**Electronic supplementary material:**

The online version of this article (10.1186/s13052-018-0467-z) contains supplementary material, which is available to authorized users.

## Background

In the last 10–15 years, the advent of high-resolution microarray technologies has revealed that cryptic chromosomal deletions and duplications, commonly defined as copy number variations (CNVs), are at the origin of a wide variety of clinical manifestations, including neurodevelopmental disorders (NDD), multiple congenital anomalies (MCA) and dysmorphic features [1].

Over time, array-based Comparative Genomic Hybridization (aCGH) has increased our knowledge about microdeletions and microduplications and a large number of novel syndromes have been characterized [2], through a “reverse dysmorphology” method [3].

Since 2010, aCGH has been the first-tier test in the diagnostic approach of children with unexplained developmental disorders or congenital anomalies [4], with a diagnostic yield of about 15% [5, 6].

The advances in molecular methodology and the broader application of aCGH led to the detection of novel pathogenic CNVs but also of numerous variants of uncertain clinical significance (VOUS).

How to appropriately interpret results of aCGH represents a challenge for the clinician especially when information found in genetic databases or scientific literature is not enough [7–10]. The clinical significance of CNVs has important implications on patient management and on family counseling, even in terms of reproductive health [11–13].

In recent years there have been several attempts to detect clinical features as predictive factors of pathogenic CNVs in patients with intellectual disability and/or multiple congenital anomalies [14–20].

The aim of the study was to analyze clinical and molecular cytogenetic data of a sample of 339 patients with NDD/MCA in order to identify whether and which of these elements could be useful to interpret unknown or poorly described rearrangements. We also set out to establish whether some core features (NDD, dysmorphisms, MCA, epilepsy) are more probably linked to pathogenic or likely pathogenic variants when isolated and in what possible combination.

Finally, we delineated a diagnostic flow-chart based on our results that could help the clinician in aCGH interpretation and the management of patients.

## Methods

We present a retrospective study on 339 patients evaluated at the Clinical Genetics Unit of Arcispedale Santa Maria Nuova, AUSL-IRCCS of Reggio Emilia. Inclusion criteria were the presence of unexplained NDD and/or MCA and/or dysmorphisms. For all patients we collected individual informed consent for the present study.

Patients were investigated through aCGH between 2005 and 2016, after signing the appropriate informed consent to genetic testing. Since 2012 aCGH have been systematically performed by 8x60K oligochips with a resolution of 100 Kb, whereas before 2012 the analysis was carried out by using different platforms and resolutions [Additional file 1: Table S1].

All data about family/clinical history and physical/dysmorphological evaluation of patients were retrospectively extracted from clinical reports. The clinical features included: family history, pre-perinatal history, neuropsychiatric evaluation, auxological parameters, minor dysmorphisms, organ malformations, neurological assessment, sensory deficits and/or anomalies of sensory organs, skeletal anomalies, joint anomalies, skin anomalies, hematologic or endocrinological diseases.

Regarding CNVs, we considered the nature of the rearrangement (deletion/duplication), the presence of multiple rearrangements, the gene content (total number of genes, disease genes, protein-encoding genes) and the presence of interrupted genes.

The clinical significance of CNVs was obtained from an accurate review of clinical reports, literature and genetic databases: UCSC Genome Browser (http://genome.ucsc.edu/) [21], DECIPHER [22], OMIM (http://www.omim.org/), NCBI (http://www.ncbi.nlm.nih.gov/pubmed/), 3D Genome browser [23]. CNVs were grouped into pathogenic and variants of uncertain significance (VOUS). The latter were further divided into likely pathogenic and likely benign.

### Statistical analysis

Descriptive statistics were used to present the data. Distribution of continuous data was assessed by Kolomogorov-Smirnov test. Categorical data, such as phenotype characteristics or somatic problems, were presented as frequencies (%) of total number of patients tested, while continuous data (number of genes and CNV size) were presented as median with interquartile range (IQR). Comparison of phenotype characteristics between two groups with negative aCGH or CNVs and between three groups of clinical significance (pathogenic, likely pathogenic and likely benign CNVs) was done using Pearson’s chi-square test or Fisher exact test. Continuous data were compared between two groups using Mann-Whitney U test and among three groups using Kruskal-Wallis test. Post-hoc analysis was applied with Bonferroni correction for all multiple comparisons. Spearman’s correlation analysis was applied to assess association of chromosome size and the number of CNVs per chromosomes. Separate logistic regression analyses were done to assess independent predictors of positive microarray and pathogenic CNVs, respectively. Variables that showed difference at *p* < 0.1 level in univariate analysis were entered into a multivariate logistic regression model and backward stepwise selection of variables was performed. Odds ratios (OR) with 95% confidence intervals (CI) were computed and the Hosmer-Lemeshow goodness-of-fit test was performed to assess overall model fit. Measures of discrimination (Nagelkerke r^2^ and area under the receiver operating characteristic curve, ROC area) were calculated for all regression models.

All statistical tests were two-sided and were performed at a 5% significance level. SPSS software (version 20.0; SPSS Inc., Chicago, IL, USA) was used for the statistical analysis.

## Results

### Clinical data

**Fig. 1:**
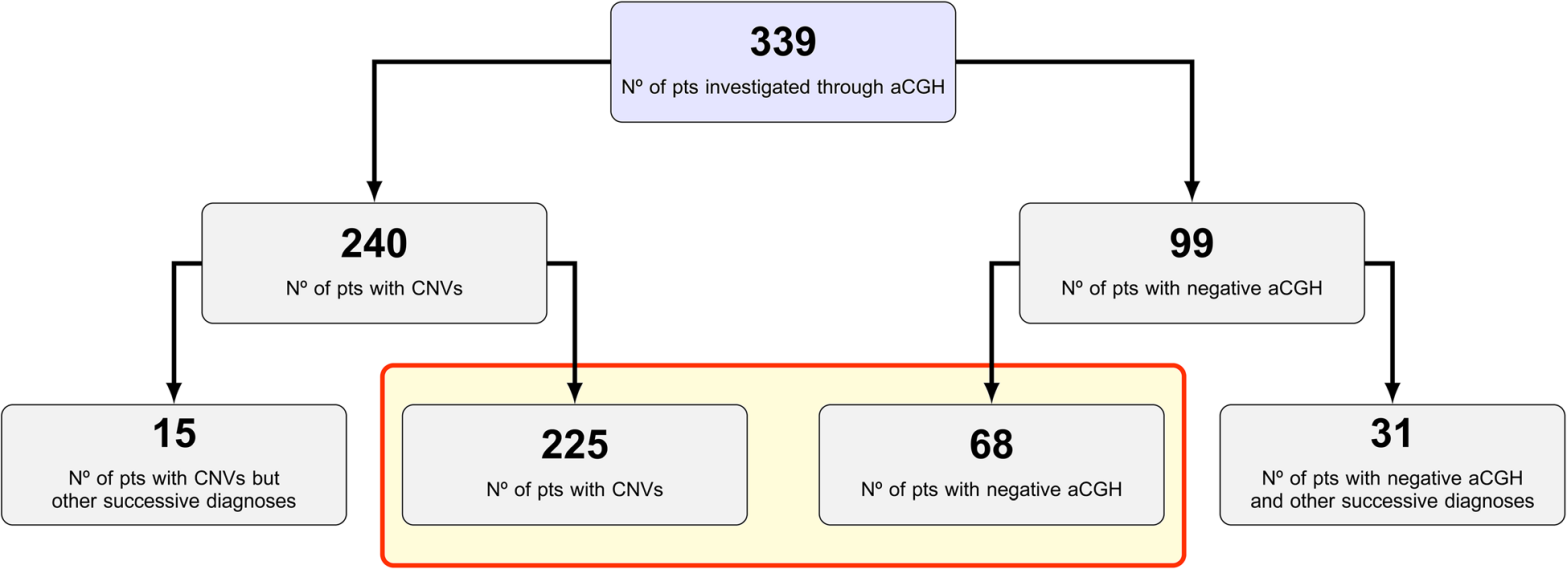
Description of the total sample enrolled investigated through aCGH until 07–31-2016. Red rectangle includes the patient group (293/339) on which the statistical analysis was performed

Of the total sample of 339 patients enrolled, 240 (70.8%) presented a genomic rearrangement (CNV), while 99 (29.2%) received a negative aCGH result. Within these two groups, some patients (15 and 31 respectively) subsequently received a different molecular or clinical diagnosis. Therefore, the final sample consisted of 293 patients (225 patients with CNVs and 68 control patients with negative aCGH) (Fig. 1).

The sample presented 169 males (57.5%) and 124 females (42.3%) with an average age at the time of the test of 7 years (range 1 month - 29 years). The majority of patients executed aCGH in the first years of life (38.5%) or at primary school age (27.6%).

Neurodevelopmental disorders (such as psychomotor developmental delay, intellectual disability, autism spectrum disorder, attention deficit and hyperactivity disorder) were observed as the main indication to perform aCGH. Psychomotor developmental delay, in particular with impairment of language (78.5%), and intellectual disability (66.4%) were the most frequently observed. 28.3% of requests concerned isolate NDD, while NDD in combination with other features (dysmorphisms, congenital malformations, epilepsy) reached 83.9%.

As regards to the severity of intellectual disability, 31.1% of the patients had mild ID, 5% of subjects with severe intellectual disability had developmental delay and language impairment, 14% of subjects presented with autism spectrum disorder.

Congenital heart anomalies (atrial septal defect, patent ductus arteriosus), ocular diseases, CNS malformations (corpus callosum, hippocampus or white matter anomalies), EEGraphic abnormalities, face and hands dysmorphisms were the most reported features [Additional file 2: Table S2].

### Molecular cytogenetic data

Among the 225 patients with CNVs, 70 (31.1%) showed pathogenic CNVs and 155 (68.8%) carried VOUS (105 likely benign and 50 likely pathogenic). Of the pathogenic CNVs, 27 were associated with known syndromes, 26 were new microdeletions or microduplications containing at least one gene whose haploinsufficiency or amplification correlates with known pathogenic conditions, and 17 were rare de novo CNVs or other chromosomal imbalances (Table 1).

**Table 1:** Pathogenic chromosomal rearrangements detected in our sample (70/293)

Known microdeletion/ microduplication syndromes or other chromosomal anomalies (27/70)	New microdeletion/ microduplication syndromes (26/70)	Rare conditions of microdeletion/ microduplication or other chromosomal anomalies (17/70)
- 22q11.2 deletion syndrome(4) - 22q11.2 distal deletion syndrome(1) - Wolf Syndrome(2) - Phelan McDermid Syndrome(2) - Di George 2 Syndrome(1) - 16p11.2 deletion syndrome(5) - Smith Magenis syndrome(1) - Xq28 duplication syndrome(3) - 1p36 deletion syndrome(2) - Turner syndrome with X-isochromosome(1) - Tetrasomy 18p(1) - Deletion Xq25 - Lowe Syndrome(1) - 1p31.1 amplification - Carney Complex(1) - Paternal UPD 14-like(1) - 11qter deletion syndrome and 9p duplication syndrome(1)	- 15q11.2 deletion syndrome(3) - 15q11.2 duplication syndrome(1) - 1q42 duplication syndrome(1) - 1q43q44 deletion syndrome(2) - 2q37 deletion syndrome(2) - 18q12.3 deletion syndrome(1) - 1q21.1 deletion syndrome(2) - 2q31.1 deletion syndrome(1) - 3q13.31 deletion syndrome(1) - 17p13.1 duplication syndrome (1) - 4q21 deletion syndrome(1) - 15q24.1 deletion syndrome(1) - 2q23.1 duplication syndrome(1) - 5q35.2q35.3 duplication syndrome(1) - 14q32.3ter deletion syndrome(1) - 16p13.11 deletion syndrome(1) - 15q11q13 duplication syndrome(1) - Xp22.31 duplication syndrome (1) - 15q13.3 deletion syndrome(1) - 8p23.1 deletion syndrome(1) - Xp11.2 duplication syndrome (1)	- Unbalanced translocation [t(7;9), t(9;10), 2 t(10;16), t(8;12)] (5) - Trip(mos)13q11q12.11 + dup(mos)13q12.11q12.3 (1) - del 7p22.3p22.2 (1) - del 15q21.3q22.2 (1) - del 18q11.2 (1) - dup 1q41q43 (1) - dup 14q11.2q12 (1) - dup 20q13.2q13.33 (1) - del 1q44 (1) - del 19q13.42q13.43 (1) - del 7q11.23q21.11 (1) - del 7q21.13q21.3 (1) - del 11q25ter (1)

*del* deletion, *dup* duplication, *mos* mosaicism, *UPD* uniparental disomy

The number of patients for each chromosomal anomaly is indicated within parentheses

In the total group of patients with chromosomal rearrangements (225), 153 (68%) presented a single CNV (73 deletions and 75 duplications), while the remaining cases had multiple rearrangements, up to 5 CNVs in a single patient. Therefore, the total number of CNVs detected were 323: 81 pathogenic, 72 VOUS likely pathogenic and 170 VOUS likely benign [Additional file 1: Table S1].

**Fig. 2:**
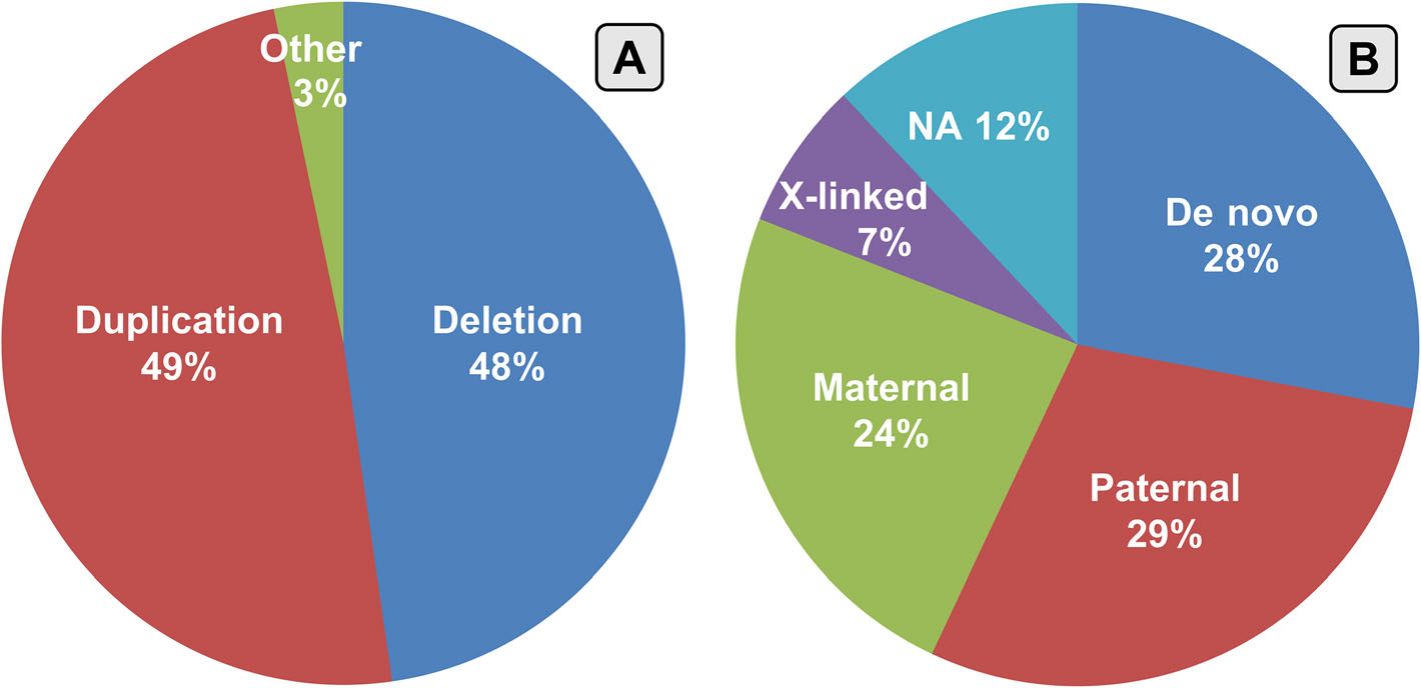
**a** percentages of CNVs distinct for type of aberrations; (**b**) percentages of CNVs distinct for hereditary pattern [NA: not available]

We detected 155 deletions and 157 duplications. In addition, we found other anomalies such as amplifications, triplications, tetrasomies or deletions and duplications in mosaic in 4.8% of the patients. 91 CNVs (28%) were de novo, 194 CNVs (60%) were inherited: autosomal inheritance from the father in 95 cases (29%), autosomal inheritance from the mother in 77 cases (24%) and X-linked transmission in 22 cases (7%). The remaining 38 CNVs (12%) were of unknown origin (Fig. 2).

The average size of the CNVs was 2494 Kb (range 18–93,000). The average number of genes located in the CNVs was 16.42 (range 0–485), of which protein coding genes 16.41 (range 0–486) and disease genes 3.58 (range 0–115).

**Fig. 3:**
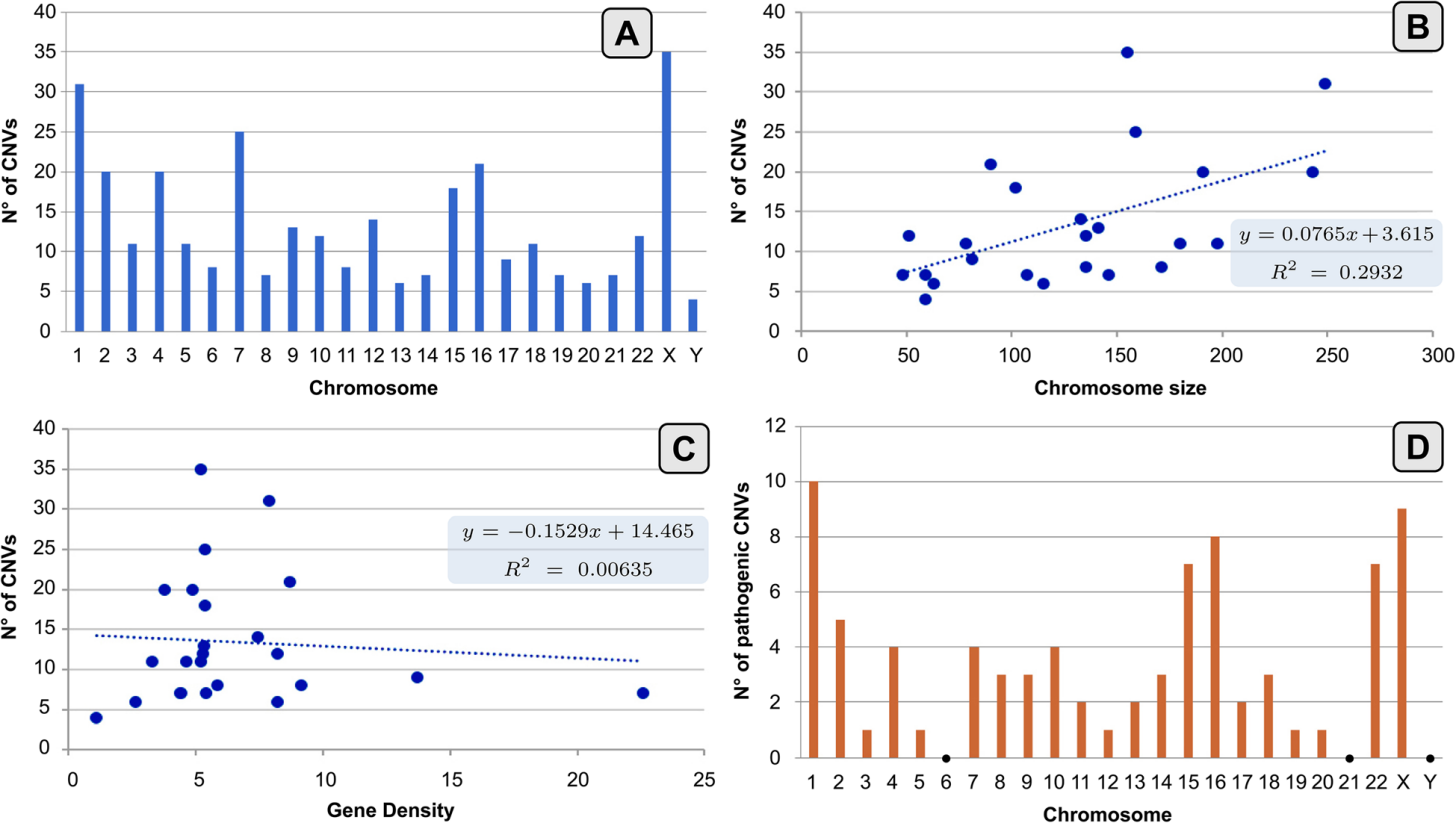
**a** CNVs distribution on chromosomes; **b** Correlation of chromosomes’ size (Mb) and number of CNVs for each chromosome; **c** Correlation of gene density and number of CNVs for each chromosome; **d** Pathogenic CNVs distribution on chromosomes

The distribution of CNVs on chromosomes did not appear to be linked to chromosome size or gene density. Notably, we observed a greater concentration of rearrangements on chromosome X (10.8%) and chromosome 1 (9.6%), but the number of CNVs did not positively correlate with the size of the chromosome (r^2^ 0.29). There was no correlation between the number of CNVs and the gene density of the chromosomes (r^2^ 0.006) (Fig. 3a-c).

Pathogenic CNVs mainly clustered on chromosomes 1, 15, 16, 22, and X (Fig. 3d).

### Data analysis

Comparing the group of patients with pathogenic CNVs and VOUS, higher statistically significant frequency (*p* < 0.01) of delayed psychomotor development, intellectual disability, IUGR, prematurity, congenital heart disease, cerebral malformations and dysmorphisms was detected in the pathogenic CNVs group. In addition, a significant difference with *p* < 0.05 for absence of speech and anomalies of the interventricular septum was found. Somatic overgrowth and autism spectrum disorders were the only two data in which a statistically significant difference (p < 0.05) was found in favor of VOUS. [Additional file 3: Table S3].

**Fig. 4:**
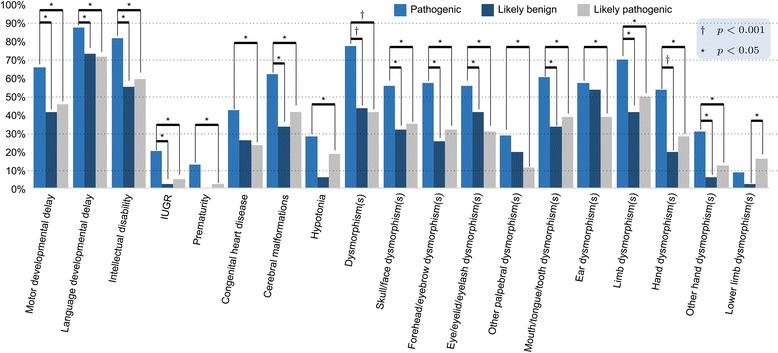
Comparison of phenotypic features between pathogenic CNVs, likely pathogenic CNVs and likely benign CNVs. Representation of variables with statistically significant difference between the three groups. Post-hoc comparison indicates to which groups this difference specifically refers. [*p < 0.05; ^†^ *p* < 0.0001]

Clinical features showing statistically significant differences among patients with pathogenic CNVs, likely pathogenic CNVs and likely benign CNVs are reported in Fig. 4 [complete description in Additional file 4: Table S4].

Lastly, we compared patients with likely pathogenic VOUS [Additional file 5: Table S5] to the group with negative aCGH (comprising likely benign VOUS plus controls). We detected statistically significant differences in favor of likely pathogenic CNVs for abnormal EEG and for limb dysmorphisms [Additional file 6: Table S6].

Multivariate logistic regression analysis of independent predictors of pathogenic CNV was consequently conducted and results are shown in Table 2. Univariate analysis showed that 27 variables were statistically significant predictors of pathogenic CNVs, of which only prematurity, ventricular septal defect (VSD) and dysmorphisms remained significant predictors of pathogenic CNVs in the multivariate logistic model. The Nagelkerke r^2^ for the final model was 0.216, the ROC area was 0.619 (95% CI 0.529–0.710; *p* < 0.013) and Hosmer-Lemeshow test for goodness of fit was statistically insignificant (*p* = 0.604).

**Table 2:** Independent predictors of pathogenic CNV

	b(SE)	OR	95%CI	*p* value
Prematurity	2.58 (0.89)	13.7	2.28–76.09	0.004
VSD	1.52 (0.75)	5.78	1.06–19.86	0.042
Dysmorphisms	1.41 (0.44)	4.09	1.73–9.66	0.001

*SE* standard error, *OR* odds ratio, *CI* confidence interval, *VSD* ventricular septal defect

As regards molecular cytogenetic characteristics, both the size of the rearrangements and the number of contained genes, protein coding genes and disease genes were statistically significant (*p* < 0.0001) for pathogenic CNVs in the comparison of the three groups (pathogenic, likely pathogenic and likely benign). The number of broken genes is not configured as a significant element. As far as inheritance is concerned, de novo CNVs were represented with statistical significance in the group of pathogenic CNVs; comparing the type of aberration, we found a greater percentage of deletions and fewer duplications in pathogenic CNVs, with statistical significance versus likely benign CNVs (Table 3).

**Table 3:** Values are median (IQR) unless otherwise stated

	Likely benign *n* = 170	Pathogenic *n* = 81	Likely pathogenic *n* = 72	Test value and overall *p* value	Comparison group	Post-hoc *p* value
Size (Kb)	234.5 (263)	2938.0 (5686)	1150.0 (3317)	*X*^2^ = 14.149 *p* < 0.0001	P vs. LB	< 0.0001
					P vs. LP	< 0.0001
					LP vs. LB	< 0.0001
Contained genes	1.5 (2)	27.0 (38)	10 (22)	*X*^2^ = 20.750 *p* < 0.0001	P vs. LB	< 0.0001
					P vs. LP	< 0.0001
					LP vs. LB	< 0.0001
Genes protein coding	2.0 (2)	29.0 (38)	11 (23)	*X*^2^ = 20.058 *p* < 0.0001	P vs. LB	< 0.0001
					P vs. LP	< 0.0001
					LP vs. LB	< 0.0001
Disease genes (morbid)	0.6 (1)	5.0 (9)	2 (4)	*X*^2^ = 15.154 *p* < 0.0001	P vs. LB	< 0.0001
					P vs. LP	< 0.0001
					LP vs. LB	< 0.0001
Interrupted genes, n (%)						
0	44 (25.9)	26 (32.1)	16 (22.2)	*X*^2^ = 2.005; *p* = 0.367	AC	NS
1	77 (45.3)	37 (45.7)	39 (54.2)	*X*^2^ = 1.721; *p* = 0.423	AC	NS
2	49 (28.8)	17 (21.0)	17 (23.6)	*X*^2^ = 1.975; *p* = 0.372	AC	NS
3	0 (0)	1 (1.2)	0 (0)	NA	AC	NS
Inheritance, n (%)						
Paternal	75 (50.0)	7 (10.0)	13 (20.6)	*X*^2^ = 40.323; *p* < 0.0001	LB vs. P	< 0.0001
					LB vs. LP	< 0.0001
					P vs. LP	NS
Maternal	67 (44.7)	6 (8.6)	20 (31.7)	*X*^2^ = 28.230 *p* < 0.0001	LB vs. P	< 0.0001
					LB vs. LP	NS
					LP vs. P	0.001
De novo	8 (5.3)	57 (81.4)	30 (47.6)	*X*^2^ = 28.230 *p* < 0.0001	P vs. LB	< 0.0001
					P vs. LP	< 0.0001
					LP vs. LB	< 0.0001
Type, n (%)						
Deletion	61 (35.9)	57 (70.4)	37 (51.4)	*X*^2^ = 26.573; *p* < 0.0001	P vs. LB	< 0.0001
					P vs. LP	NS
					LP vs. LB	NS
Duplication	103 (60.6)	22 (27.1)	32 (44.4)	*X*^2^ = 25.182; *p* < 0.0001	LB vs. P	< 0.0001
					P vs. LP	NS
					LP vs. LB	NS
Others	6 (3.5)	2 (2.5)	3 (4.2)	*X*^2^ = 0.351; *p* = 0.839	AC	NS

*X*^2^ Pearson’s or Kruskal Wallis Chi-Square test, *LB* likely benign, *P* pathogenic, *LP* likely pathogenic, *AC* all post-hoc comparisons, *NA* not applicable, *NS* not significant

In addition, for those patients (*n* = 16) who had a single CNV of uncertain significance and not containing any known protein coding genes, we performed an in silico prediction of the noncoding elements and of the possible modification of topologically associating domains (TADs) by consulting the 3D Genome Browser [23] [Additional file 7: Table S7]. This analysis provided some useful insights on CNVs previously dismissed as non-significant, suggesting a novel functional approach that might be included in the current interpretation guidelines.

Then we analyzed the core features (NDD/Dysmorphisms/MCA/Epilepsy) for which aCGH was performed in each patient, as either isolated or associated elements. Comparing these core features with the results of aCGH, we observed that it was more likely to find an abnormal rearrangement when NDD were associated with other features rather than isolated. In patients with NDD alone we observed a statistically significant presence of negative aCGH (*p* = 0.0003) compared to presence of CNVs, while in patients with a combination of NDD and dysmorphisms we found a statistically significant presence of CNVs (*p* = 0.0358) [Additional file 8: Table S8]. Specifically, in patients with isolated NDD and presence of CNVs we observed likely benign CNVs more frequently than pathogenic or likely pathogenic CNVs (*p* < 0.00001); whereas in subjects with NDD associated with dysmorphism, pathogenic CNVs were more likely to be detected (*p* = 0.0042) (Table 4).

**Table 4:** Correlations between phenotypical core features and aCGH results (pathogenic CNVs vs likely pathogenic CNVs vs likely benign CNVs)

	pathogenic (*N* = 70)		likely pathogenic (*N* = 50)		likely benign (*N* = 105)		*p* value
	Number	Percent	Number	Percent	Number	Percent	
NDD	2	2.86	12	24	38	36.19	**< 0.00001**
Dysmorphism(s)	0	0.00	1	2	1	0.95	NA
MCA	5	7.14	1	2	8	7.62	0.371509
Epilepsy	0	0.00	2	4	1	0.95	NA
NDD + Dysmorphism(s)	25	35.71	13	26	15	14.29	***0.004246***
NDD + MCA	10	14.29	8	16	12	11.43	0.70822
NDD + epilepsy	1	1.43	4	8	9	8.57	0.13395
NDD + dysmorphism(s) + MCA	12	17.14	4	8	10	9.52	0.20385
NDD + dysmorphism(s) + epilepsy	2	2.86	1	2	1	0.95	0.640556
NDD + MCA + epilepsy	4	5.71	2	4	3	2.86	0.823368
NDD + dysmorphism(s) + MCA + epilespy	2	2.86	0	0	0	0.00	NA
Dysmorphism(s) + MCA	5	7.14	2	4	4	3.81	0.573256
Dysmorphism(s) + epilepsy	0	0.00	0	0	0	0.00	NA
MCA + epilepsy	2	2.86	0	0	2	1.90	NA
Dysmorphism(s) + MCA + epilepsy	0	0.00	0	0	0	0.00	NA
Other	0	0.00	0	0	1	0.95	NA

*MCA* multiple congenital anomalies, *NDD* neurodevelopmental disorders

Results significant for likely benign CNVs (bold); results significant for pathogenic CNVs (bold and italic)

Regarding NDD reported in our sample, individuals with moderate to severe ID were around 20% (59/293) of total patients [Additional file 2: Table S2] and the rate of pathogenic CNVs in this group was 28.8% (17/59). Accordingly, next generation sequencing analysis identified single nucleotide variants in 39% of patients with severe intellectual disability while causative CNVs in only 21% of them [24].

### Suggested diagnostic flow-chart

**Fig. 5:**
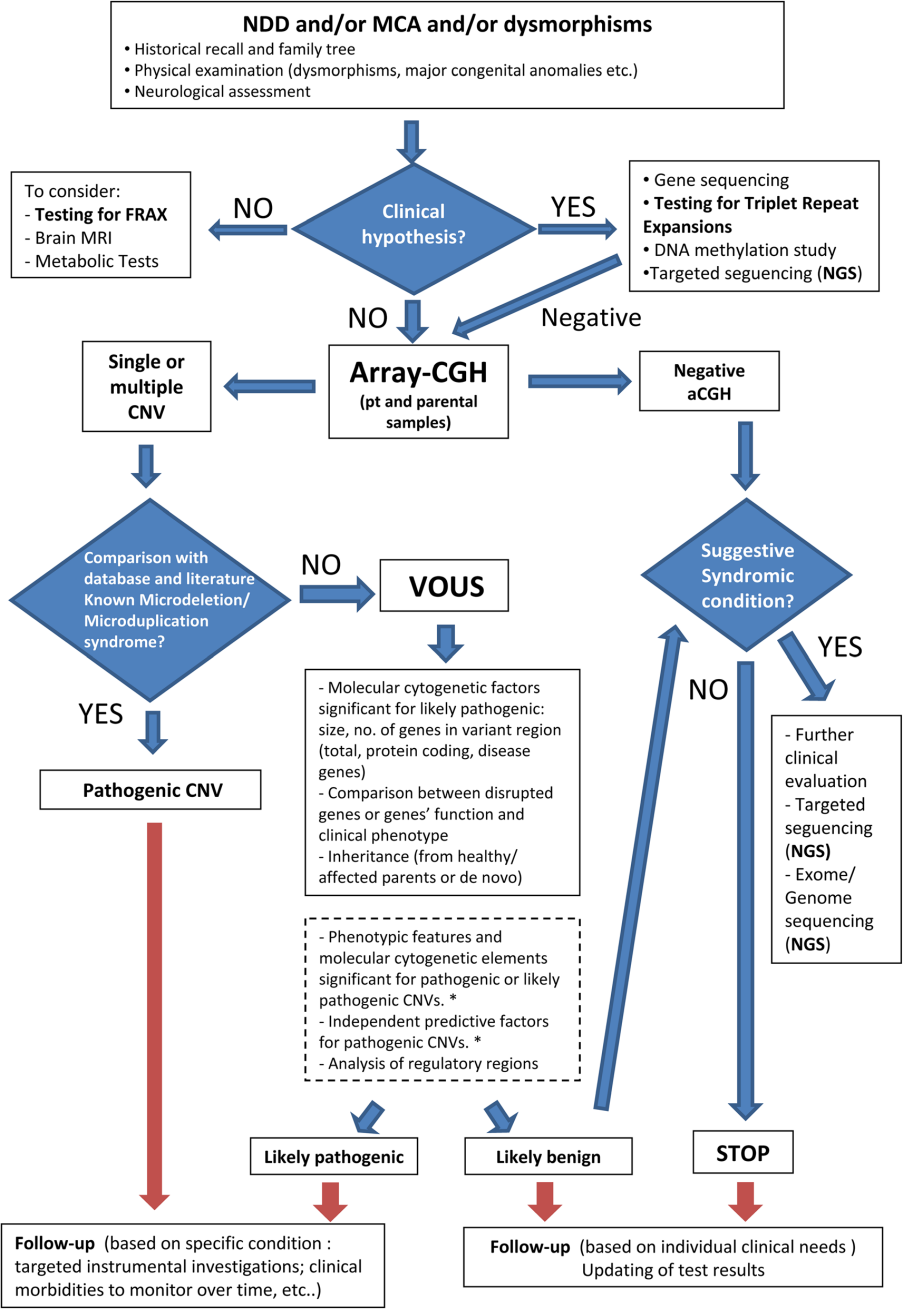
Flow-chart in patients with NDD and/or MCA and/or Dysmorphisms. The first step is the collection of appropriate family and clinical history and physical/dysmorphological evaluation. If the patient has a recognizable syndrome, we have to confirm it with specific genetic tests. Otherwise, except for other possible neurological or metabolic implications, we will proceed by considering aCGH (in case of male subjects with ID, it would be appropriate to consider the molecular survey for Fragile X syndrome). The blood draw should always be done on the trio in order to perform aCGH on parent’s sample if anomalous in the child. If aCGH detects CNVs, they will be carefully interpreted. Some CNVs can be classified as pathogenic because linked to known syndromes or to “new microdeletion/microduplication syndromes”. If CNVs are less known or poorly described they have an uncertain clinical significance (VOUS): we suggest some variables that might be useful in distinguishing likely pathogenic from likely benign CNVs (continuous box). Additionally, the presence of some phenotypic variables, as well as the analysis of non-coding regions, could be useful in classifying VOUS as likely pathogenic (dashed box) [* Phenotypic variables significant for pathogenic CNVs: developmental delay, ID, prematurity, IUGR, dysmorphisms, congenital heart disease, hypotonia, cerebral malformations; Phenotypic variables significant for likely pathogenic CNVs: abnormal EEG, hand and lower limb dysmorphisms; Independent predictive factors for pathogenic CNVs: prematurity, ventricular septal defect, dysmorphisms]. In the case of normal chromosomal pattern or likely benign CNVs, it will be necessary to re-evaluate the patient. If the clinical features are strongly suggestive of a genetic/syndromic condition further genetic investigations will be carried out. These may include targeted sequencing, exome sequencing and, in selected cases, genome sequencing. Otherwise clinical follow up should be implemented in the event that evocative elements could emerge over time recommending future genetic investigations

A flow-chart (Fig. 5) regarding the care for patients with NDD and/or MCA and/or dysmorphisms has been made on the basis of the data inferred from this study and their comparison with the literature [14–20]. Our purpose is to make the investigative process of CNVs more informative and to suggest possible guide elements in aCGH interpretation. It is of primary importance because patient follow-up and reproductive counseling to families could change considerably depending on the meaning of aCGH results.

## Discussion

In the last 10–15 years, aCGH has been a revolutionary tool in identifying genomic aberrations in the broad spectrum of pediatric population with neurodevelopmental disorders and/or multiple congenital anomalies [4, 25], modifying the management of these patients and their families [6, 13].

In the literature, the detection rate of pathogenic CNVs in these patients ranges from 5 to 20% (on average 15%), depending on the preselection of patients and on the technical characteristics of the instrument used [26, 27].

In our study of 339 patients with NDD and/or MCA tested by aCGH, the detection rate of pathogenic CNVs was 20.6% (70/339). This result could be representative of an appropriate selection of the patients who underwent this genetic test in our clinical unit.

In our sample, 55.6% (163/293) of patients presented with NDD associated with MCA and/or dysmorphisms and/or epilepsy, while 28.3% (83/293) had an isolated NDD. The residual percentage of patients had malformative, dysmorphic or neurological characteristics, isolated or in combination with each other [Additional file 7: Table S7]. The detection of pathogenic CNVs in case of isolated NDD was extremely low (2/293, 0.68%), while it reached 19.1% (56/293) in cases of NDD associated with other clinical elements. In particular, we found pathogenic CNVs in 8.5% (25/293) of subjects with NDD and dysmorphisms (Table 4).

In recent years, wide use of aCGH in patients with NDD and/or MCA has led to the detection of an impressive number of VOUS.

The interpretation of aCGH results became a crucial topic in clinical practice, in diagnostic, prognostic and ethical terms. Thus, recently, several studies focused on identifying specific clinical or phenotype variables that could be associated with the detection of pathogenic CNVs (Table 5).

**Table 5:** Partial review of literature on detection of phenotypic factors related to pathogenic CNVs in patients with NDD and/or MCA

References	N° pt	Main Phenotype	Technique	Detection rate pathogenic CNVs	Clinical variables associated to pathogenic CNVs	Independent predictors of pathogenic CNVs
Caballero Pérez et al. [15]	80	DD, ID	–	27.5%	- Positive family history for DD/ID - Malformations - > n° 3 dysmorphisms - Hypotonia	–
Cappuccio et al. [14]	214	ID, ASD, M	Oligo (500Kb e 50-75Kb)	30%	- ASD - Positive family history for ID/ASD/MCA	- ID - Positive family history for ID - cutaneous dyschromia
Preiksaitiene et al. [17]	211	DD, ID	Oligo 44 K, 400 K, 105 K SNP 300 K, 700 K (−)	13.7%	- Cerebral malformations (CC) - Hydrocephalus - Dysmorphisms (down slanting palpebral fissures, ears, micrognathia) - Brachydactyly -Umbilical hernia - “coffee and milk” spots	- Congenital anomalies of corpus callosus - Ear dysmorphisms - Brachydactyly
Caramaschi et al. [18]	116	DD, ID + E/ M/D	Oligo 44 K (−)	23.3%	- Early onset symptoms (< 1 y) - Dysmorphisms - Malformations	- Dysmorphisms - Malformations
D’Arrigo et al. [16]	329	DD, ID	Oligo 4x180K (40 Kb)	16%	- Positive family history for DD/ID - IUGR - Face Dysmorphisms	- Positive family history for DD/ID - IUGR - Facies Dysmorphisms
Shoukier et al. [20]	342	DD, ID, M	Oligo 244 K 4x180K	13.2%	- Congenital anomalies (heart)	–
Roselló et al. [19]	246	DD, ID + M, D	BAC (0.5–1 Mb) Oligo 44 K (50–150 Kb)	29.7%	- Somatic overgrowth - Dysmorphisms (low set ears, hypertelorism, II-V finger anomalies) - Genital anomalies - VSD	–
Our study	339	DD, ID, ASD, M, D	Oligo 6x80K (100 Kb)^a^	20.6%	- DD/ID - Prematurity, IUGR - Hypotonia - Congenital heart anomalies - Cerebral malformations - Face and hand dysmorphisms	- Prematurity - VSD - Dysmorphisms

*ASD* autism spectrum disorder, *D* dysmorphisms, *DD* developmental delay, *ID* intellectual disability, *E* epilepsy, *M* malformations

^a^technique and resolution most commonly used in the sample of our study

In our study, the univariate analysis detected a statistically significant association of pathogenic CNVs (vs likely pathogenic CNVs and likely benign CNVs) with different variables summarized in Fig. 4, only partially described in previous studies [14–20].

Moreover, prematurity, dysmorphisms and interventricular septal defect resulted as independent predictors of pathogenic CNVs.

Prematurity has not been previously reported. Premature subjects, who survive the neonatal period, are characterized by a high risk of developing NDD. In light of this data, it could be interesting to consider prematurity as a phenotypic feature within the syndromic frame caused by pathogenic CNVs.

VSD, as well as congenital heart anomalies in general, have already been described as linked to pathogenic CNVs [19]. In our study, VSD assumed an independent predictive value for pathogenic CNVs. This data appears to be supported by recent literature, which reports significant CNVs in 16.9% of patients with VSD [28].

Finally, dysmorphisms play an important predictive role for pathogenic CNVs and present a statistically significant association with pathogenic CNVs when considered in association with NDD.

Regarding molecular cytogenetic characteristics, we found that pathogenicity is significantly correlated with the larger size of aberrations, the greater number of total/protein-coding/disease genes located within, the de novo mode of inheritance and the deletion type of variants. These elements have already been described [10, 29]. We also detected abnormal EEG, hand dysmorphisms and lower limb dysmorphisms as more frequent variables in likely pathogenic CNVs versus likely benign CNVs plus negative aCGH. Clinically, these data may have modest impact, but comparisons between these two groups of patients had not been previously described in literature.

Moreover, the presence of disrupted genes and the study of gene function compared to patient phenotype could provide important clues for the interpretation of CNVs. Likewise, the analysis of TADs appears to have a predictive value, also for the evaluation of likely benign VOUS (Fig. 5) [Additional file 7: Table S7].

We are aware that the molecular cytogenetic and phenotypic elements identified may not assume an absolute value in the interpretation of results, but they could contribute to the framework needed for the clinician in discerning between likely pathogenic and likely benign VOUS (Fig. 5).

The main limit of the study is that we did not use standardized criteria in classifying patients’ CNVs (pathogenic, likely pathogenic and likely benign), because there are no specific references in the literature. The study was retrospective and patients were divided into the three categories of significance on the basis of their clinical reports. In any case, CNV interpretation has been the result of careful analysis of scientific literature, genetic database and phenotype evaluation.

## Conclusions

In our retrospective analysis, we observed a detection rate of pathogenic CNVs at the upper limits of what was reported in literature [26, 27]. It could be the result of a careful selection of patients that underwent aCGH in our clinical unit.

In patients with NDD, prematurity is usually considered as an environmental risk factor. In our study, the detection of prematurity as an independent predictor of pathogenic CNVs suggests that sometimes this feature can rather be considered as a main part of the underlying genetic disorder.

Dysmorphisms, especially if associated with NDD, seem to have a predictive significance for pathogenic aberrations.

We detected several elements related to pathogenic CNVs and some related to likely pathogenic CNVs that could be helpful in the interpretation of aCGH results, even though we acknowledge they may not assume an absolute significance in the interpretative process. This necessarily requires a combination of several factors, such as scientific literature, genetic databases, molecular cytogenetic characteristics, detailed patient anamnesis and phenotype evaluation.

However, it is necessary to emphasize the importance of a meticulous description of the phenotypic features of patients with pathogenic CNVs, both to contribute to scientific sharing of data and to facilitate accurate interpretation of aCGH results [17].

The purpose of the study was to improve diagnostic accuracy, with a positive impact on patients’ clinical management, prognosis, follow-up and genetic counseling.

## Additional files

Additional file 1: **Table S1.** All CNVs of the analyzed sample (323 CNVs). Molecular cytogenetic data of all CNVs detected (type, position, number of genes, inheritance), technical characteristics of microarray performed (platform and resolution) and clinical significance assigned to CNV for the single patient [ampl: amplification, del: deletion, dup: duplication, tetr: tetrasomy, trip: triplication, *: mosaicism, mat: maternal, pat: paternal, NA: not available, P: pathogenic, LB: likely benign, LP: likely pathogenic]. (XLS 95 kb)

Additional file 2: **Table S2** Clinical and Phenotypic features of the analyzed sample (293 patients). Total number and percentage as compared to the number of patients for which the single data was available. [ADHD: attention deficit and hyperactivity disorder, ASD: atrial septal defect, CNS: central nervous system, IUGR: intrauterine growth retardation, PDA: patent ductus arteriosus, ToF: Tetralogy of Fallot, VSD: ventral septal defect]. (DOC 90 kb)

Additional file 3: **Table S3.** Correlations between clinical and phenotypic features and aCGH results (pathogenic CNVs vs VOUS). Statistically significant results for pathogenic CNVs are reported in bold and significant data for VOUS are reported in italics. [ADHD: Attention deficit and hyperactivity disorder; ASD: atrial septal defect; CNS: central nervous system; CTG: fetal cardiotocography; IUGR: intrauterine growth restriction; PDA: patent ductus arteriosus; PFO: patent foramen ovale; ToF: Tetralogy of Fallot; VSD: interventricular septal defect]. (DOC 163 kb)

Additional file 4: **Table S4.** Correlations between clinical and phenotypic features and aCGH results (pathogenic CNVs vs likely pathogenic CNVs vs likely benign CNVs). All statistically significant features are reported in bold. From post-hoc analysis with Bonferroni correction: *Significant in the comparison of pathogenic and likely benign; §Significant in the comparison of pathogenic and likely pathogenic; ¥Significant in the comparison of likely pathogenic and likely benign. [ADHD: Attention deficit and hyperactivity disorder; ASD: atrial septal defect; CNS: central nervous system; CTG: fetal cardiotocography; IUGR: intrauterine growth restriction; PDA: patent ductus arteriosus; PFO: patent foramen ovale; ToF: Tetralogy of Fallot; VSD: ventricular septal defect]. (DOC 194 kb)

Additional file 5: **Table S5.** Molecular cytogenetic and phenotypic data of patients with likely pathogenic CNVs (50 patients). [del: deletion, dup: duplication, mat: maternal, pat: paternal, NA: not available, LB: likely benign, LP: likely pathogenic; ID: intellectual disability; ASD: autism spectrum disorder; ADHD: attention deficit hyperactivity disorder; NDD: neurodevelopmental disorders; CHD: congenital heart defect]. (XLS 90 kb)

Additional file 6: **Table S6.** Correlations between clinical and phenotypic features and aCGH results (likely pathogenic VOUS vs likely benign VOUS + negative aCGH). Statistically significant results for likely pathogenic VOUS are reported in bold [n/N, number of cases with positive variable/number of patients with available data on that variable; NA: not applicable; ADHD: Attention deficit and hyperactivity disorder; ASD: atrial septal defect; CNS: central nervous system; CTG: fetal cardiotocography; IUGR: intrauterine growth restriction; PDA: patent ductus arteriosus; PFO: patent foramen ovale; ToF: Tetralogy of Fallot; VSD: interventricular septal defect]. (DOC 170 kb)

Additional file 7: **Table S7.** In silico analysis of single CNVs falling in gene-desert regions. Prediction of the influence of CNVs on topologically associating domains (TADs), discrete genomic regions characterized by a high frequency of self-interaction. The presence of noncoding, potentially regulatory elements and the possible positional effect of alterations are also considered. [lincRNA: long intergenic noncoding RNA; H3K27Ac, H3K4Me1: Histone 3 acetilation/methylation, may indicate active regulatory elements; ^a^ across TAD boundary; ^b^ within TAD]. (XLS 27 kb)

Additional file 8: **Table S8.** Correlations between phenotypical core features and aCGH results (positive aCGH vs negative aCGH). Statistically significant results for negative aCGH are reported in bold; statistically significant results for positive aCGH are reported in bold and italic. [MCA: multiple congenital anomalies; NDD: neurodevelopmental disorders]. (DOC 49 kb)

## Abbreviations

aCGH: array-CGH
ASD: Autism Spectrum Disorder
CNVs: Copy Number Variations
ID: Intellectual disability
MCA: Multiple Congenital Anomalies
NDD: Neurodevelopmental Disorders
VOUS: Variant of uncertain significance

## References

[1] Rosenfeld JA, Patel A (2017). Chromosomal microarrays: understanding genetics of neurodevelopmental disorders and congenital anomalies. J Pediatr Genet.

[2] Nevado J, Mergener R, Palomares-Bralo M, Souza KR, Vallespín E, Mena R (2014). New microdeletion and microduplication syndromes: a comprehensive review. Genet Mol Biol.

[3] Slavotinek AM (2008). Novel microdeletion syndromes detected by chromosome microarrays. Hum Genet.

[4] Miller DT, Adam MP, Aradhya S, Biesecker LG, Brothman AR, Carter NP (2010). Consensus statement: chromosomal microarray is a first-tier clinical diagnostic test for individuals with developmental disabilities or congenital anomalies. Am J Hum Genet.

[5] Chong WW, Lo IF, Lam ST, Wang CC, Luk HM, Leung TY (2014). Performance of chromosomal microarray for patients with intellectual disabilities/developmental delay, autism, and multiple congenital anomalies in a Chinese cohort. Mol Cytogenet.

[6] Riggs ER, Wain KE, Riethmaier D, Smith-Packard B, Faucett WA, Hoppman N (2014). Chromosomal microarray impacts clinical management. Clin Genet.

[7] Nowakowska B (2017). Clinical interpretation of copy number variants in the human genome. J Appl Genetics.

[8] Palmer E, Speirs H, Taylor PJ, Mullan G, Turner G, Einfeld S (2014). Changing interpretation of chromosomal microarray over time in a community cohort with intellectual disability. Am J Med Genet A.

[9] Hehir-Kwa JY, Pfundt R, Veltman JA, de Leeuw N (2013). Pathogenic or not? Assessing the clinical relevance of copy number variants. Clin Genet.

[10] Gijsbers AC, Schoumans J, Ruivenkamp CA (2011). Interpretation of array comparative genome hybridization data: a major challenge. Cytogenet Genome Res.

[11] Torres F, Barbosa M, Maciel P (2016). Recurrent copy number variations as risk factors for neurodevelopmental disorders: critical overview and analysis of clinical implication. J Med Genet.

[12] Tao VQ, Chan KYK, Chu YWY, Mok GTK, Tan TY, Yang W, et al. The clinical impact of chromosomal microarray on paediatric care in Hong Kong. PLoS One. 2014; 10.1371/journal.pone.0109629. 10.1371/journal.pone.0109629 PMC4198120 25333781

[13] Coulter ME, Miller DT, Harris DJ, Hawley P, Picker J, Roberts AE (2011). Chromosmal microarray testing influences medical management. Genet Med.

[14] Cappuccio G, Vitiello F, Casertano A, Fontana P, Genesio R, Bruzzese D (2016). New insights in the interpretation of array-CGH: autism spectrum disorder and positive family history for intellectual disability predict the detection of pathogenic variants. Ital J Pediric.

[15] Caballero Pérez V, López Pisón FJ, Miramar Gallart MD, González Álvarez A, García Jiménez MC, García Iñiguez JP, et al. Phenotype in patients with intellectual disability and pathological results in array CGH. Neurologia. 2016; 10.1016/j.nrl.2016.03.006. 10.1016/j.nrl.2016.03.006 27157524

[16] D'Arrigo S, Gavazzi F, Alfei E, Zuffardi O, Montomoli C, Corso B (2016). The diagnostic yield of Array comparative genomic hybridization is high regardless of severity of intellectual disability/developmental delay in children. J Child Neurol.

[17] Preiksaitiene E, Molytė A, Kasnauskiene J, Ciuladaite Z, Utkus A, Patsalis PC (2014). Considering specific clinical features as evidence of pathogenic copy number variants. J Appl Genet.

[18] Caramaschi E, Stanghellini I, Magini P, Giuffrida MG, Scullin S, Giuva T (2014). Predictive diagnostic value for the clinical features accompanying intellectual disability in children with pathogenic copy number variations: a multivariate analysis. Ital J Pediatr.

[19] Roselló M, Martínez F, Monfort S, Mayo S, Oltra S, Orellana C (2014). Phenotype profiling of patients with intellectual disability and copy number variations. Eur J Paediatr Neurol.

[20] Shoukier M, Klein N, Auber B, Wickert J, Schröder J, Zoll B (2013). Array CGH in patients with developmental delay or intellectual disability: are there phenotypic clues to pathogenic copy number variants?. Clin Genet.

[21] Kent WJ, Sugnet CW, Furey TS, Roskin KM, Pringle TH, Zahler AM (2002). The human genome browser at UCSC. Genome Res.

[22] Firth HV, Richards SM, Bevan AP, Clayton S, Corpas M, Rajan D (2009). DECIPHER: database of chromosomal imbalance and phenotype in humans using Ensembl resources. Am J Hum Genet.

[23] Wang Y, Zhang B, Zhang L, An L, Xu J, Li D. The 3D genome browser: a web-based browser for visualizing 3D genome organization and long-range chromatin interactions. BioRxiv. 2017; 10.1101/112268. 10.1186/s13059-018-1519-9 PMC6172833 30286773

[24] Gilissen C, Hehir-Kwa JY, Thung DT, van de Vorst M, van Bon BW, Willemsen MH (2014). Genom sequencing identifies major causes of severe intellectual disability. Nature.

[25] Beaudet AL (2013). The utility of chromosomal microarray analysis in developmental and behavioral pediatrics. Child Dev.

[26] Cooper GM, Coe BP, Girirajan S, Rosenfeld JA, Vu TH, Baker C (2011). A copy number variation morbidity map of developmental delay. Nat Genet.

[27] Kaminsky EB, Kaul V, Paschall J, Church DM, Bunke B, Kunig D (2011). An evidence-based approach to establish the functional and clinical significance of copy number variants in intellectual and developmental disabilities. Genet Med..

[28] An Y, Duan W, Huang G, Chen X, Li L, Nie C (2016). Genome-wide copy number variant analysis for congenital ventricular septal defects in Chinese Han population. BMC Med Genet.

[29] Vulto-van Silfhout AT, Hehir-Kwa JY, van Bon BW, Schuurs-Hoeijmakers JH, Meader S (2013). Clinical significance of de novo and inherited copy-number variation. Hum Mutat.

