# Supplementary material for: Prematurity, ventricular septal defect and dysmorphisms are independent predictors of pathogenic copy number variants: a retrospective study on array-CGH results and phenotypical features of 293 children with neurodevelopmental disorders and/or multiple congenital anomalies
Source: Ital J Pediatr. 2018 Mar 9;44:34. doi: 10.1186/s13052-018-0467-z (PMC5845186; doi:10.1186/s13052-018-0467-z)
Supplement: Supplementary file 2 — Table S2 Clinical and Phenotypic features of the analyzed sample (293 patients). Total number and percentage as compared to the number of patients for which the single data was available. [ADHD: attention deficit and hyperactivity disorder, ASD: atrial septal defect, CNS: central nervous system, IUGR: intrauterine growth retardation, PDA: patent ductus arteriosus, ToF: Tetralogy of Fallot, VSD: ventral septal defect]. (DOC 90 kb) [file 13052_2018_467_MOESM2_ESM.doc]

**Table S2 - Clinical and Phenotypic features of the analyzed sample (293 patients)**

| **Features** | **Frequency** | **%** |
| --- | --- | --- |
| *Neuropsychiatric features* |  |  |
| Motor developmental delay | 147/293 | 50.7 |
| Language developmental delay | 227/293 | 78.5 |
| Intellectual disability (ID)  mild ID  moderate ID  severe ID | 152/293  91/293  51/293  8/293 | 66.4  31.1  17.4  2.7 |
| Learning disorders | 16/293 | 5.6 |
| Language disorder | 69/293 | 24 |
| Absent speech | 41/293 | 14.4 |
| Autism spectrum disorder | 40/293 | 13.9 |
| ADHD | 7/293 | 2.4 |
| Behavioral disorders | 33/293 | 11.5 |
| Psychiatric illness | 1/293 | 0.4 |
|  |  |  |
| *Clinical features* |  |  |
| IUGR | 28/292 | 9.6 |
| Prematurity | 11/228 | 4.8 |
| Macrocephaly | 49/292 | 16.8 |
| Microcephaly | 43/292 | 14.7 |
| Short stature | 56/292 | 19.2 |
| Overgrowth | 16/292 | 5.5 |
| Congenital heart disease | 78/234 | 33.3 |
| ASD | 19/234 | 8.1 |
| VSD | 21/234 | 9 |
| PDA | 24/234 | 10.3 |
| Patent foramen ovale | 17/234 | 7.3 |
| ToF | 2/234 | 0.9 |
| Aortic valve anomalies | 6/234 | 2.6 |
| Pulmonary valve anomalies | 9/234 | 3.8 |
| Mitral valve anomalies | 10/234 | 4.3 |
| Other cardiac anomalies | 26/234 | 11.1 |
| Respiratory malformations | 17/292 | 5.8 |
| Kidney malformations | 36/292 | 12.3 |
| Gastroenteric malformations | 38/292 | 13 |
| Genital malformations | 25/292 | 8.6 |
| Cryptorchidism | 15/292 | 5.1 |
| Hypospadias | 3/292 | 1 |
| Other genital anomalies | 9/292 | 3.1 |
| CNS malformations | 131/293 | 44.7 |
| Corpus callosum anomalies | 36/293 | 12.3 |
| White matter anomalies | 18/293 | 6.1 |
| Hippocampus anomalies | 23/293 | 6.8 |
| Other CNS anomalies | 109/293 | 37.2 |
| Epilepsy | 50/287 | 17.4 |
| EEG anoamalies | 108/287 | 37.6 |
| Neurological anomalies | 123/292 | 42.1 |
| Dyspraxia | 16/292 | 5.5 |
| Hypotonia | 53/292 | 18.2 |
| Clumsiness | 22/292 | 7.5 |
| Other neurological anomalies | 66/292 | 22.6 |
| Hearing loss | 32/234 | 13.7 |
| Sensorineural hearing loss | 10/234 | 4.3 |
| Conductive hearing loss | 17/234 | 7.3 |
| Ocular anomalies | 102/233 | 43.8 |
| Astigmatism | 20/233 | 8.6 |
| Myopia | 13/233 | 5.6 |
| Hypermetropia | 18/233 | 7.7 |
| Strabismus | 59/233 | 25.3 |
| Knee anomalies | 34/291 | 11.7 |
| Scoliosis | 33/293 | 11.3 |
| Flat feet | 28/293 | 9.6 |
| Other skeletal anomalies | 45/293 | 15.4 |
| Delayed bone age | 4/293 | 1.4 |
| Advanced bone age | 3/293 | 1 |
| Hypothyroidism | 12/293 | 4.1 |
| Obesity | 7/293 | 2.4 |
| GH deficiency | 4/293 | 1.4 |
|  |  |  |
| *Dysmorphic features* |  |  |
| Dysmorphisms | 154/292 | 52.7 |
| Skull/face | 124/292 | 42.5 |
| Forehead/eyebrows | 107/292 | 36.6 |
| Eyes/eyelids/eyelashes | 121/292 | 41.4 |
| Hypertelorism | 20/292 | 6.8 |
| Epicanthus | 33/292 | 11.3 |
| Up-slanting palpebral fissures | 31/292 | 10.6 |
| Down-slanting palpebral fissures | 18/292 | 6.2 |
| Nose | 97/292 | 33.2 |
| Philtrum | 40/292 | 13.7 |
| Mouth/teeth/tongue | 135/292 | 46.2 |
| Ears | 141/292 | 48.3 |
| Neck/chest | 33/292 | 11.3 |
| Limbs | 160/292 | 54.8 |
| Hands | 108/293 | 36.9 |
| Hand brachydactyly | 15/293 | 5.1 |
| Hand clinodactyly | 37/292 | 12.6 |
| Hand syndactyly | 1/293 | 0.3 |
| Hand camptodactyly | 8/293 | 2.7 |
| Arachnodactyly | 17/293 | 5.8 |
| Foot syndactyly | 14/293 | 4.8 |
| Hair anomalies | 26/293 | 8.9 |
| Nails anomalies | 18/293 | 6.1 |
| Skin anomalies | 76/293 | 25.9 |
| Skin softness | 27/293 | 9.2 |
| Joint laxity | 44/293 | 15 |

**Total number and percentage as compared to the number of patients for which the single data was available. [ADHD: attention deficit and hyperactivity disorder, ASD: atrial septal defect, CNS: central nervous system, ID: intellectual disability, IUGR: intrauterine growth retardation, PDA: patent ductus arteriosus, ToF: Tetralogy of Fallot, VSD: ventricular septal defect]**
