# Supplementary material for: Prematurity, ventricular septal defect and dysmorphisms are independent predictors of pathogenic copy number variants: a retrospective study on array-CGH results and phenotypical features of 293 children with neurodevelopmental disorders and/or multiple congenital anomalies
Source: Ital J Pediatr. 2018 Mar 9;44:34. doi: 10.1186/s13052-018-0467-z (PMC5845186; doi:10.1186/s13052-018-0467-z)
Supplement: Supplementary file 3 — Table S3. Correlations between clinical and phenotypic features and aCGH results (pathogenic CNVs vs VOUS). Statistically significant results for pathogenic CNVs are reported in bold and significant data for VOUS are reported in italics. [ADHD: Attention deficit and hyperactivity disorder; ASD: atrial septal defect; CNS: central nervous system; CTG: fetal cardiotocography; IUGR: intrauterine growth restriction; PDA: patent ductus arteriosus; PFO: patent foramen ovale; ToF: Tetralogy of Fallot; VSD: interventricular septal defect]. (DOC 163 kb) [file 13052_2018_467_MOESM3_ESM.doc]

**Table S3 - Correlations between clinical and phenotypical features and aCGH results (pathogenic CNVs vs VOUS)**

|  | **Total**  **(N = 225)** | | **Pathogenic CNV**  **(N = 70)** | | **VOUS**  **(N = 155)** | | **p value** |
| --- | --- | --- | --- | --- | --- | --- | --- |
|  | n | % | n | % | n | % |  |
| **Motor developmental delay** | 115 | 51.6 | 46 | 66.7 | 69 | 44.8 | **0.003** |
| **Language developmental delay** | 173 | 77.6 | 60 | 88.2 | 113 | 72.9 | **0.001** |
| Language disorder | 60 | 27.1 | 17 | 25.4 | 43 | 27.9 | 0.695 |
| **Absent speech** | 32 | 14.4 | 15 | 22.4 | 17 | 11.0 | **0.026** |
| **Intellectual disability** | 117 | 65.7 | 43 | 82.7 | 74 | 58.7 | **0.002** |
| Learning disorders | 13 | 5.8 | 2 | 2.9 | 11 | 7.1 | 0.353 |
| Autism spectrum disorders | 31 | 14.0 | 4 | 6.0 | 27 | 17.4 | **0.024** |
| ADHD | 4 | 1.8 | 1 | 1.5 | 3 | 1.9 | 0.820 |
| Behavioral disorders | 28 | 12.6 | 12 | 17.9 | 16 | 10.3 | 0.118 |
| Psychiatric illness | 0 | 0.0 | 0 | 0.0 | 0 | 0.0 | NA |
| Positive family history | 167 | 75.2 | 52 | 76.5 | 115 | 74.7 | 0.775 |
| Consanguinity | 11 | 4.9 | 3 | 4.3 | 8 | 5.2 | 0.778 |
| **IUGR** | 20 | 8.9 | 14 | 20.3 | 6 | 3.9 | **<0,001** |
| Fetal/perinatal distress | 3 | 1.7 | 1 | 2.2 | 2 | 1.5 | 0.765 |
| Neonatal hypotonia | 1 | 0.6 | 1 | 2.2 | 0 | 0.0 | 0.258 |
| **Prematurity** | 8 | 4.5 | 6 | 13.0 | 2 | 1.5 | **0.004** |
| CTG anomalies | 1 | 0.6 | 1 | 2.1 | 0 | 0.0 | 0.263 |
| Respiratory distress | 1 | 0.6 | 0 | 0.0 | 1 | 0.8 | 0.555 |
| Macrocephaly | 36 | 16.1 | 11 | 15.9 | 25 | 16.1 | 0.972 |
| Microcephaly | 30 | 13.4 | 8 | 11.6 | 22 | 14.2 | 0.598 |
| Short stature | 41 | 18.3 | 14 | 20.3 | 27 | 17.4 | 0.608 |
| *Overgrowth* | 10 | 4.5 | 0 | 0.0 | 10 | 6.5 | *0.034* |
| **Congenital heart disease** | 59 | 30.9 | 28 | 43.1 | 31 | 24.6 | **0.009** |
| ASD | 15 | 7.9 | 8 | 12.3 | 7 | 5.6 | 0.100 |
| **VSD** | 16 | 8.4 | 9 | 13.8 | 7 | 5.6 | **0.050** |
| PDA | 19 | 9.9 | 10 | 15.4 | 9 | 7.1 | 0.071 |
| Patent foramen ovale | 12 | 6.3 | 4 | 6.2 | 8 | 6.3 | 1.000 |
| ToF | 1 | 0.5 | 0 | 0.0 | 1 | 0.8 | 1.000 |
| Aortic valve anomalies | 4 | 2.1 | 2 | 3.1 | 2 | 1.6 | 0.606 |
| Pulmonary valve anomalies | 5 | 2.6 | 2 | 3.1 | 3 | 2.4 | 1.000 |
| Mitral valve anomalies | 7 | 3.7 | 3 | 4.6 | 4 | 3.2 | 0.691 |
| Other cardiac anomalies | 18 | 9.4 | 9 | 13.8 | 9 | 7.1 | 0.133 |
| Respiratory malformations | 12 | 5.4 | 6 | 8.6 | 6 | 3.9 | 0.199 |
| Kidney malformations | 29 | 12.9 | 11 | 15.7 | 18 | 11.7 | 0.405 |
| Gastroenteric malformations | 30 | 13.4 | 13 | 18.6 | 17 | 11.0 | 0.125 |
| Genital malformations | 20 | 8.9 | 7 | 10.0 | 13 | 8.4 | 0.705 |
| Cryptorchidism | 12 | 5.4 | 4 | 5.7 | 8 | 5.2 | 1.000 |
| Hypospadias | 3 | 1.3 | 1 | 1.4 | 2 | 1.3 | 1.000 |
| Other genital anomalies | 7 | 3.1 | 2 | 2.9 | 5 | 3.2 | 0.877 |
| **CNS malformations** | 105 | 46.7 | 44 | 62.9 | 61 | 39.4 | **0.001** |
| Corpus callosum anomalies | 30 | 28.0 | 14 | 31.8 | 16 | 25.4 | 0.467 |
| White matter anomalies | 13 | 12.1 | 6 | 13.6 | 7 | 11.1 | 0.694 |
| Hippocampus anomalies | 20 | 18.7 | 10 | 22.7 | 10 | 15.9 | 0.371 |
| Other CNS anomalies | 88 | 82.2 | 34 | 77.3 | 54 | 85.7 | 0.261 |
| Epilepsy | 42 | 19.2 | 12 | 17.6 | 30 | 19.9 | 0.699 |
| EEG anomalies | 91 | 41.6 | 30 | 44.1 | 61 | 40.4 | 0.605 |
| Neurological anomalies | 99 | 44.2 | 36 | 51.4 | 63 | 40.9 | 0.142 |
| Dyspraxia | 13 | 5.8 | 5 | 7.1 | 8 | 5.2 | 0.550 |
| **Hypotonia** | 43 | 19.2 | 20 | 28.6 | 23 | 14.9 | **0.016** |
| Clumsiness | 17 | 7.6 | 4 | 5.7 | 13 | 8.4 | 0.593 |
| Other neurological anomalies | 53 | 23.7 | 15 | 21.4 | 38 | 24.7 | 0.596 |
| Hearing loss | 26 | 14.2 | 10 | 16.4 | 16 | 13.1 | 0.549 |
| Sensorineural hearing loss | 8 | 4.4 | 5 | 8.2 | 3 | 2.5 | 0.119 |
| Conductive hearing loss | 13 | 7.1 | 4 | 6.6 | 9 | 7.4 | 0.839 |
| Other hearing anomalies | 8 | 4.3 | 2 | 3.3 | 6 | 4.9 | 0.617 |
| Ocular anomalies | 75 | 39.9 | 30 | 47.6 | 45 | 36.0 | 0.125 |
| Astigmatism | 10 | 5.3 | 4 | 6.3 | 6 | 4.8 | 0.734 |
| Myopia | 9 | 4.8 | 3 | 4.8 | 6 | 4.8 | 1.000 |
| Hypermetropia | 14 | 7.4 | 6 | 9.5 | 8 | 6.4 | 0.557 |
| Strabismus | 49 | 26.1 | 17 | 27.0 | 32 | 25.6 | 0.838 |
| Exophoria | 5 | 2.7 | 0 | 0.0 | 5 | 4.0 | 0.170 |
| Exotropia | 12 | 6.4 | 6 | 9.5 | 6 | 4.8 | 0.221 |
| Esotropia | 18 | 9.6 | 3 | 4.8 | 15 | 12.0 | 0.111 |
| Other ocular anomalies | 17 | 9.0 | 8 | 12.7 | 9 | 7.2 | 0.221 |
| **Dysmorphisms** | 120 | 53.6 | 54 | 78.3 | 66 | 42.6 | **<0.001** |
| **Skull/face** | 92 | 41.1 | 39 | 56.5 | 53 | 34.2 | **0.002** |
| **Forehead/eyebrows** | 87 | 38.8 | 40 | 58.0 | 47 | 30.3 | **<0.001** |
| **Eyes/eyelids/eyelashes** | 93 | 41.5 | 39 | 56.5 | 54 | 34.8 | **0.002** |
| Hypertelorism | 19 | 8.5 | 8 | 11.6 | 11 | 7.1 | 0.265 |
| Epicanthus | 24 | 10.7 | 8 | 11.6 | 16 | 10.3 | 0.776 |
| Up-slanting palpebral fissures | 26 | 11.6 | 10 | 14.5 | 16 | 10.3 | 0.368 |
| Down-slanting palpebral fissures | 13 | 5.8 | 4 | 5.8 | 9 | 5.8 | 1.000 |
| **Other eye dysmorphisms** | 42 | 18.8 | 20 | 29.0 | 22 | 14.2 | **0.009** |
| **Nose** | 73 | 32.6 | 30 | 43.5 | 43 | 27.7 | **0.002** |
| Philtrum | 31 | 13.8 | 11 | 15.9 | 20 | 12.9 | 0.543 |
| **Mouth/teeth/tongue** | 100 | 44.6 | 42 | 60.9 | 58 | 37.4 | **0.001** |
| Ears | 108 | 48.2 | 40 | 58.0 | 68 | 43.9 | 0.051 |
| Neck/chest | 24 | 10.7 | 10 | 14.5 | 14 | 9.0 | 0.223 |
| **Limbs** | 123 | 54.9 | 49 | 71.0 | 74 | 47.7 | **0.001** |
| **Hands** | 78 | 34.7 | 38 | 54.3 | 40 | 25.8 | **<0.001** |
| Hand brachydactyly | 11 | 4.9 | 4 | 5.7 | 7 | 4.5 | 0.743 |
| Hand clinodactyly | 26 | 11.6 | 11 | 15.7 | 15 | 9.7 | 0.190 |
| Hand syndactyly | 1 | 0.4 | 0 | 0.0 | 1 | 0.6 | 1.000 |
| Hand camptodactyly | 5 | 2.2 | 1 | 1.4 | 4 | 2.6 | 0.587 |
| Arachnodactyly | 11 | 4.9 | 5 | 7.1 | 6 | 3.9 | 0.324 |
| **Other hand dysmorphisms** | 38 | 16.9 | 22 | 31.4 | 16 | 10.3 | **<0.001** |
| Flat feet | 23 | 10.2 | 8 | 11.4 | 15 | 9.7 | 0.688 |
| Foot syndactyly | 12 | 5.3 | 4 | 5.7 | 8 | 5.2 | 1.000 |
| Other foot dysmorphisms | 56 | 24.9 | 21 | 30.0 | 35 | 22.6 | 0.233 |
| Lower limb dysmorphisms | 24 | 10.8 | 6 | 8.7 | 18 | 11.7 | 0.505 |
| Scoliosis | 25 | 11.1 | 12 | 17.1 | 13 | 8.4 | 0.053 |
| Other skeletal dysmorphisms | 32 | 14.2 | 10 | 14.3 | 22 | 14.2 | 1.000 |
| Hair dysmorphisms | 19 | 8.4 | 5 | 7.1 | 14 | 9.0 | 0.637 |
| Nail dysmoprhisms | 15 | 6.7 | 5 | 7.1 | 10 | 6.5 | 0.847 |
| Skin anomalies | 57 | 25.3 | 20 | 28.6 | 37 | 23.9 | 0.453 |
| Delayed bone age | 2 | 0.9 | 2 | 2.9 | 0 | 0.0 | 0.096 |
| Advanced bone age | 2 | 0.9 | 0 | 0.0 | 2 | 1.3 | 0.340 |
| Hypothyroidism | 9 | 4.0 | 3 | 4.3 | 6 | 3.9 | 0.883 |
| Obesity | 4 | 1.8 | 3 | 4.3 | 1 | 0.6 | 0.056 |
| GH deficiency | 4 | 1.8 | 3 | 4.3 | 1 | 0.6 | 0.056 |
| Other endocrinological anomalies | 12 | 5.3 | 5 | 7.1 | 7 | 4.5 | 0.522 |
| Skin softness | 17 | 7.6 | 6 | 8.6 | 11 | 7.1 | 0.708 |
| Joint laxity | 217 | 96.4 | 66 | 94.3 | 151 | 97.4 | 0.259 |

Statistically significant results for pathogenic CNVs are reported in bold and significant data for VOUS are reported in italics. Results are reported in italics and significant data for positive aCGH are reported in bold. [ADHD: Attention deficit and hyperactivity disorder; ASD: atrial septal defect; CNS: central nervous system; CTG: fetal cardiotocography; IUGR: intrauterine growth restriction; PDA: patent ductus arteriosus; PFO: patent foramen ovale; ToF: Tetralogy of Fallot; VSD: interventricular septal defect]
