# Supplementary material for: Prematurity, ventricular septal defect and dysmorphisms are independent predictors of pathogenic copy number variants: a retrospective study on array-CGH results and phenotypical features of 293 children with neurodevelopmental disorders and/or multiple congenital anomalies
Source: Ital J Pediatr. 2018 Mar 9;44:34. doi: 10.1186/s13052-018-0467-z (PMC5845186; doi:10.1186/s13052-018-0467-z)
Supplement: Supplementary file 4 — Table S4. Correlations between clinical and phenotypic features and aCGH results (pathogenic CNVs vs likely pathogenic CNVs vs likely benign CNVs). All statistically significant features are reported in bold. From post-hoc analysis with Bonferroni correction: *Significant in the comparison of pathogenic and likely benign; §Significant in the comparison of pathogenic and likely pathogenic; ¥Significant in the comparison of likely pathogenic and likely benign. [ADHD: Attention deficit and hyperactivity disorder; ASD: atrial septal defect; CNS: central nervous system; CTG: fetal cardiotocography; IUGR: intrauterine growth restriction; PDA: patent ductus arteriosus; PFO: patent foramen ovale; ToF: Tetralogy of Fallot; VSD: ventricular septal defect]. (DOC 194 kb) [file 13052_2018_467_MOESM4_ESM.doc]

**Table S4 - Correlations between clinical and phenotypical features and aCGH results (pathogenic CNVs vs likely pathogenic CNVs vs likely benign CNVs)**

|  | Total  (N =225) | | | Likely benign CNV  (N = 105) | | Pathogenic CNV  (N = 70) | | Likely pathogenic CNV  (N = 50) | | p value |
| --- | --- | --- | --- | --- | --- | --- | --- | --- | --- | --- |
|  | n | % | | n | % | n | % | n | % |  |
| **Motor development delay** | 115 | 51.6 | | 48 | 46.2 | 46 | 66.7 | 21 | 42.0 | **0.009*^§^** |
| **Language development delay** | 173 | 77.6 | | 76 | 72.4 | 60 | 88.2 | 37 | 74.0 | **0.040*^§^** |
| Language disorder | 60 | 27.1 | | 27 | 25.7 | 17 | 25.4 | 16 | 32.7 | 0.617 |
| Absent speech | 32 | 14.4 | | 13 | 12.4 | 15 | 22.4 | 4 | 8.0 | 0.065 |
| **Intellectual disability** | 117 | 65.7 | | 50 | 60.2 | 43 | 82.7 | 24 | 55.8 | **0.008*^§^** |
| Learning disorders | 13 | 5.8 | | 8 | 7.6 | 2 | 2.9 | 3 | 6.0 | 0.439 |
| Autism spectrum disorders | 31 | 14.0 | | 18 | 17.1 | 4 | 6.0 | 9 | 18.0 | 0.077 |
| ADHD | 4 | 1.8 | | 3 | 2.9 | 1 | 1.5 | 0 | 0.0 | 0.446 |
| Behavioral disorders | 28 | 12.6 | | 11 | 10.5 | 12 | 17.9 | 5 | 10.0 | 0.294 |
| Psychiatric illness | 0 | 0.0 | | 0 | 0.0 | 0 | 0.0 | 0 | 0.0 | NA |
| Positive family history | 167 | 75.2 | | 82 | 788 | 52 | 76.5 | 33 | 66.0 | 0.215 |
| Consanguinity | 11 | 4.9 | | 7 | 6.7 | 3 | 4.3 | 1 | 2.0 | 0.435 |
| **IUGR** | 20 | 8.9 | | 5 | 4.8 | 14 | 20.3 | 1 | 2.0 | **<0.001*^§^** |
| Fetal/perinatal distress | 3 | 1.7 | | 2 | 2.2 | 1 | 2.2 | 0 | 0.0 | 0.624 |
| Neonatal hypotonia | 1 | 0.6 | | 0 | 0.0 | 1 | 2.2 | 0 | 0.0 | 0.236 |
| **Prematurity** | 8 | 4.5 | | 2 | 2.2 | 6 | 13.0 | 0 | 0.0 | **0.004*** |
| CTG anomalies | 1 | 0.6 | | 0 | 0.0 | 1 | 2.1 | 0 | 0.0 | 0.244 |
| Respiratory distress | 1 | 0.6 | | 1 | 1.1 | 0 | 0.0 | 0 | 0.0 | 0.615 |
| Macrocephaly | 36 | 16.1 | | 19 | 18.1 | 11 | 15.9 | 6 | 12.0 | 0.627 |
| Microcephaly | 30 | 13.4 | | 16 | 15.2 | 8 | 11.6 | 6 | 12.0 | 0.747 |
| Short stature | 41 | 18.3 | | 18 | 17.1 | 14 | 20.3 | 9 | 18.0 | 0.869 |
| Overgrowth | 10 | 4.5 | | 6 | 5.7 | 0 | 0.0 | 4 | 8.0 | 0.079 |
| **Congenital heart disease** | 59 | 30.9 | | 21 | 23.9 | 28 | 43.1 | 10 | 26.3 | **0.031*** |
| ASD | 15 | 7.9 | | 5 | 5.7 | 8 | 12.3 | 2 | 5.3 | 0.258 |
| VSD | 16 | 8.4 | | 4 | 4.5 | 9 | 13.8 | 3 | 7.9 | 0.121 |
| PDA | 19 | 9.9 | | 7 | 8.0 | 10 | 15.4 | 2 | 5.3 | 0.177 |
| Patent foramen ovale | 12 | 6.3 | | 5 | 5.7 | 4 | 6.2 | 3 | 7.9 | 0.894 |
| ToF | 1 | 0.5 | | 1 | 1.1 | 0 | 0.0 | 0 | 0.0 | 0.555 |
| Aortic valve anomalies | 4 | 2.1 | | 2 | 2.3 | 2 | 3.1 | 0 | 0.0 | 0.568 |
| Pulmonary valve anomalies | 5 | 2.6 | | 2 | 2.3 | 2 | 3.1 | 1 | 2.6 | 0.954 |
| Mitral valve anomalies | 7 | 3.7 | | 2 | 2.3 | 3 | 4.6 | 2 | 5.3 | 0.630 |
| Other cardiac anomalies | 18 | 9.4 | | 4 | 4.5 | 9 | 13.8 | 5 | 13.2 | 0.102 |
| Respiratory malformations | 12 | 5.4 | | 4 | 3.8 | 6 | 8.6 | 2 | 4.1 | 0.354 |
| Kidney malformations | 29 | 12.9 | | 10 | 9.5 | 11 | 15.7 | 8 | 16.3 | 0.356 |
| Gastroenteric malformations | 30 | 13.4 | | 12 | 11.4 | 13 | 18.6 | 5 | 10.2 | 0.302 |
| Genital malformations | 20 | 8.9 | | 7 | 6.7 | 7 | 10.0 | 6 | 12.2 | 0.491 |
| Cryptorchidism | 12 | 5.4 | | 4 | 3.8 | 4 | 5.7 | 4 | 8.2 | 0.529 |
| Hypospadias | 3 | 1.3 | | 2 | 1.9 | 1 | 1.4 | 0 | 0.0 | 0.630 |
| Other genital anomalies | 7 | 3.1 | | 2 | 1.9 | 2 | 2.9 | 3 | 6.1 | 0.370 |
| **CNS malformations** | 105 | 46.7 | | 44 | 41.9 | 44 | 62.9 | 17 | 34.0 | **0.003*^§^** |
| Corpus callosum anomalies | 30 | 28.0 | | 13 | 29.5 | 14 | 31.8 | 3 | 15.8 | 0.412 |
| White matter anomalies | 13 | 12.1 | | 5 | 11.4 | 6 | 13.6 | 2 | 10.5 | 0.922 |
| Hippocampus anomalies | 20 | 18.7 | | 7 | 15.9 | 10 | 22.7 | 3 | 15.8 | 0.670 |
| Other CNS anomalies | 88 | 82.2 | | 35 | 79.5 | 34 | 77.3 | 19 | 100.0 | 0.079 |
| Epilepsy | 42 | 19.2 | | 19 | 18.4 | 12 | 17.6 | 11 | 22.9 | 0.752 |
| EEG anomalies | 91 | 41.6 | | 37 | 35.9 | 30 | 44.1 | 24 | 50.0 | 0.230 |
| Neurological anomalies | 99 | 44.2 | | 48 | 45.7 | 36 | 51.4 | 15 | 30.6 | 0.072 |
| Dyspraxia | 13 | 5.8 | | 6 | 5.7 | 5 | 7.1 | 2 | 4.1 | 0.780 |
| **Hypotonia** | 43 | 19.2 | | 20 | 19.0 | 20 | 28.6 | 3 | 6.1 | **0.009^§^** |
| Clumsiness | 17 | 7.6 | | 8 | 7.6 | 4 | 5.7 | 5 | 10.2 | 0.661 |
| Other neurological anomalies | 53 | 23.7 | | 27 | 25.7 | 15 | 21.4 | 11 | 22.4 | 0.787 |
| Hearing loss | 26 | 14.2 | | 11 | 13.8 | 10 | 16.4 | 5 | 11.9 | 0.804 |
| Sensorineural hearing loss | 8 | 4.4 | | 1 | 1.2 | 5 | 8.2 | 2 | 4.8 | 0.134 |
| Conductive hearing loss | 13 | 7.1 | | 6 | 7.5 | 4 | 6.6 | 3 | 7.1 | 0.977 |
| Other hearing anomalies | 8 | 4.3 | | 6 | 7.4 | 2 | 3.3 | 0 | 0.0 | 0.142 |
| Ocular anomalies | 75 | 39.9 | | 31 | 37.3 | 30 | 47.6 | 14 | 33.3 | 0.280 |
| Astigmatism | 10 | 5.3 | | 3 | 3.6 | 4 | 6.3 | 3 | 7.1 | 0.641 |
| Myopia | 9 | 4.8 | | 4 | 4.8 | 3 | 4.8 | 2 | 4.8 | 1.000 |
| Hypermetropia | 14 | 7.4 | | 6 | 7.2 | 6 | 9.5 | 2 | 4.8 | 0.657 |
| Strabismus | 49 | 26.1 | | 23 | 27.7 | 17 | 27.0 | 9 | 21.4 | 0.736 |
| Exophoria | 5 | 2.7 | | 3 | 3.6 | 0 | 0.0 | 2 | 4.8 | 0.255 |
| Exotropia | 12 | 6.4 | | 3 | 3.6 | 6 | 9.5 | 3 | 7.1 | 0.342 |
| Esotropia | 18 | 9.6 | | 12 | 14.5 | 3 | 4.8 | 3 | 7.1 | 0.119 |
| Other ocular anomalies | 17 | 9.0 | | 7 | 8.4 | 8 | 12.7 | 2 | 4.8 | 0.368 |
| **Dysmorphisms** | 120 | 53.6 | | 44 | 41.9 | 54 | 78.3 | 22 | 44.0 | **<0.001*^§^** |
| **Skull/face** | 92 | 41.1 | | 37 | 35.2 | 39 | 56.5 | 16 | 32.0 | **0.007*^§^** |
| **Forehead/eyebrows** | 87 | 38.8 | | 34 | 32.4 | 40 | 58.0 | 13 | 26.0 | **<0.001*^§^** |
| **Eyes/eyelids/eyelashes** | 93 | 41.5 | | 33 | 31.4 | 39 | 56.5 | 21 | 42.0 | **0.005*** |
| Hypertelorism | 19 | 8.5 | | 7 | 6.7 | 8 | 11.6 | 4 | 8.0 | 0.516 |
| Epicanthus | 24 | 10.7 | | 10 | 9.5 | 8 | 11.6 | 6 | 12.0 | 0.862 |
| Up-slanting palpebral fissures | 26 | 11.6 | | 9 | 8.6 | 10 | 14.5 | 7 | 14.0 | 0.410 |
| Down-slanting palpebral fissures | 13 | 5.8 | | 8 | 7.6 | 4 | 5.8 | 1 | 2.0 | 0.376 |
| **Other eye dysmorphisms** | 42 | 18.8 | | 12 | 11.4 | 20 | 29.0 | 10 | 20.0 | **0.014*** |
| Nose | 73 | 32.6 | | 30 | 28.6 | 30 | 43.5 | 13 | 26.0 | 0.064 |
| Philtrum | 31 | 13.8 | | 16 | 15.2 | 11 | 15.9 | 4 | 8.0 | 0.395 |
| **Mouth/teeth/tongue** | 100 | 44.6 | | 41 | 39.0 | 42 | 60.9 | 17 | 34.0 | **0.004*^§^** |
| **Ears** | 108 | 48.2 | | 41 | 39.0 | 40 | 58.0 | 27 | 54.0 | **0.033*** |
| Neck/chest | 24 | 10.7 | | 8 | 7.6 | 10 | 14.5 | 6 | 12.0 | 0.338 |
| **Limbs** | 123 | 54.9 | | 53 | 50.5 | 49 | 71.0 | 21 | 42.0 | **0.003*^§^** |
| **Hands** | 78 | 34.7 | | 30 | 28.6 | 38 | 54.3 | 10 | 20.0 | **<0.001*^§^** |
| Hand brachydactyly | 11 | 4.9 | | 4 | 3.8 | 4 | 5.7 | 3 | 6.0 | 0.779 |
| Hand clinodactyly | 26 | 11.6 | | 12 | 11.4 | 11 | 15.7 | 3 | 6.0 | 0.260 |
| Hand syndactyly | 1 | 0.4 | | 1 | 1.0 | 0 | 0.0 | 0 | 0.0 | 0.563 |
| Hand camptodactyly | 5 | 2.2 | | 4 | 3.8 | 1 | 1.4 | 0 | 0.0 | 0.278 |
| Arachnodactyly | 11 | 4.9 | | 3 | 2.9 | 5 | 7.1 | 3 | 6.0 | 0.401 |
| **Other hand dysmorphisms** | 38 | 16.9 | | 13 | 12.4 | 22 | 31.4 | 3 | 6.0 | **<0.001*^§^** |
| Flat feet | 23 | 10.2 | | 9 | 8.6 | 8 | 11.4 | 6 | 12.0 | 0.743 |
| Foot syndactyly | 12 | 5.3 | | 6 | 5.7 | 4 | 5.7 | 2 | 4.0 | 0.893 |
| Other foot dysmorphisms | 56 | 24.9 | | 24 | 22.9 | 21 | 30.0 | 11 | 22.0 | 0.488 |
| **Lower limb dysmorphisms** | 24 | 10.8 | | 17 | 16.3 | 6 | 8.7 | 1 | 2.0 | **0.021^¥^** |
| Scoliosis | 25 | 11.1 | | 11 | 10.5 | 12 | 17.1 | 2 | 4.0 | 0.075 |
| Other skeletal dysmorphisms | 32 | 14.2 | | 17 | 16.2 | 10 | 14.3 | 5 | 10.0 | 0.587 |
| Hair dysmorphisms | 19 | 8.4 | | 11 | 10.5 | 5 | 7.1 | 3 | 6.0 | 0.577 |
| Nail dysmorhisms | 15 | 6.7 | | 8 | 7.6 | 5 | 7.1 | 2 | 4.0 | 0.687 |
| Skin anomalies | 57 | 25.3 | | 27 | 25.7 | 20 | 28.6 | 10 | 20.0 | 0.563 |
| Delayed bone age | 2 | 0.9 | | 0 | 0.0 | 2 | 2.9 | 0 | 0.0 | 0.107 |
| Advanced bone age | 2 | 0.9 | | 2 | 1.9 | 0 | 0.0 | 0 | 0.0 | 0.316 |
| Hypothyroidism | 9 | 4.0 | | 5 | 4.8 | 3 | 4.3 | 1 | 2.0 | 0.707 |
| Obesity | 4 | 1.8 | | 1 | 1.0 | 3 | 4.3 | 0 | 0,0 | 0.147 |
| GH deficiency | 4 | 1.8 | | 1 | 1.0 | 3 | 4.3 | 0 | 0.0 | 0.147 |
| Other endocrinological anomalies | 12 | 5.3 | | 5 | 4.8 | 5 | 7.1 | 2 | 4.0 | 0.705 |
| Skin softness | 17 | 7.6 | | 9 | 8.6 | 6 | 8.6 | 2 | 4.1 | 0.577 |
| Joint laxity | 217 | 96.4 | 102 | | 97.1 | 66 | 94.3 | 49 | 98.0 | 0.483 |

All statistically significant elements are reported In bold from post-hoc analysis with Bonferroni correction:
* Significant in the comparison of likely benign and pathogenic;
§ Significant in the comparison between pathogenic and likely pathogenic;
¥ significant in the comparison of likely benign and likely pathogenic.
[ADHD: Attention deficit and hyperactivity disorder; ASD: atrial septal defect; CNS: central nervous system; CTG: fetal cardiotocography; IUGR: intrauterine growth restriction; PDA: patent ductus arteriosus; PFO: patent foramen ovale; TOF: tetralogy of Fallot; VSD: interventricular septal defect]
