# Supplementary material for: Prematurity, ventricular septal defect and dysmorphisms are independent predictors of pathogenic copy number variants: a retrospective study on array-CGH results and phenotypical features of 293 children with neurodevelopmental disorders and/or multiple congenital anomalies
Source: Ital J Pediatr. 2018 Mar 9;44:34. doi: 10.1186/s13052-018-0467-z (PMC5845186; doi:10.1186/s13052-018-0467-z)
Supplement: Supplementary file 6 — Table S6. Correlations between clinical and phenotypic features and aCGH results (likely pathogenic VOUS vs likely benign VOUS + negative aCGH). Statistically significant results for likely pathogenic VOUS are reported in bold [n/N, number of cases with positive variable/number of patients with available data on that variable; NA: not applicable; ADHD: Attention deficit and hyperactivity disorder; ASD: atrial septal defect; CNS: central nervous system; CTG: fetal cardiotocography; IUGR: intrauterine growth restriction; PDA: patent ductus arteriosus; PFO: patent foramen ovale; ToF: Tetralogy of Fallot; VSD: interventricular septal defect]. (DOC 170 kb) [file 13052_2018_467_MOESM6_ESM.doc]

**Table S6 - Correlations between clinical and phenotypical features and aCGH results (likely pathogenic VOUS vs likely benign VOUS + negative aCGH)**

|  | Likely pathogenic  N = 50 | | Likely benign + negative aCGH  N = 173 | |  |
| --- | --- | --- | --- | --- | --- |
|  |  |
|  | n/N | % | n /N | % | P value |
| Motor developmental delay | 21/50 | 42.0 | 80/171 | 46.8 | 0.550 |
| Language developmental delay | 37/50 | 74.0 | 130/171 | 76.0 | 0.770 |
| Language disorder | 16/49 | 32.7 | 36/171 | 21.1 | 0.092 |
| Absent language | 4/50 | 8.0 | 22/168 | 13.1 | 0.329 |
| Intellectual disability | 24/43 | 55.8 | 85/134 | 63.4 | 0.371 |
| Learning disorders | 3/50 | 6.0 | 11/170 | 6.5 | 0.905 |
| Autism spectrum disorders | 9/50 | 18.0 | 27/170 | 15.9 | 0.772 |
| ADHD | 0/50 | 0.0 | 6/170 | 3.5 | 0.341 |
| Behavioral disorders | 5/50 | 10.0 | 16/170 | 9.4 | 0.901 |
| Psychiatric disease | 0/48 | 0.0 | 1/167 | 0.6 | 1.000 |
| Positive family history | 33/50 | 66.0 | 129/172 | 75.0 | 0.207 |
| Consanguinity | 1/50 | 2.0 | 10/173 | 5.8 | 0.463 |
| IUGR | 1/50 | 2.0 | 13/173 | 7.5 | 0.201 |
| Fetal/perinatal distress | 0/42 | 0.0 | 6/138 | 4.3 | 0.338 |
| Neonatal hypotonia | 0/42 | 0.0 | 0/138 | 0.0 | NA |
| Prematurity | 0/42 | 0.0 | 5/140 | 3.6 | 0.591 |
| CTG anomalies | 0/42 | 0.0 | 1/139 | 0.7 | 0.581 |
| Respiratory distress | 0/42 | 0.0 | 1/139 | 0.7 | 1.000 |
| Macrocephaly | 6/50 | 12.0 | 32/173 | 18.5 | 0.282 |
| Microcephaly | 6/50 | 12.0 | 29/173 | 16.8 | 0.415 |
| Short stature | 9/50 | 18.0 | 33/173 | 9.1 | 0.864 |
| Overgrowth | 4/50 | 8.0 | 12/173 | 6.9 | 0.761 |
| Congenital heart disease | 10/38 | 26.3 | 40/131 | 30.5 | 0.616 |
| ASD | 2/38 | 5.3 | 9/131 | 6.9 | 0.724 |
| VSD | 3/38 | 7.9 | 9/131 | 6.9 | 0.829 |
| PDA | 2/38 | 5.3 | 12/131 | 9.2 | 0.738 |
| Patent foramen ovale | 3/38 | 7.9 | 10/131 | 7.6 | 0.958 |
| ToF | 0/38 | 0.0 | 2/131 | 1.5 | 1.000 |
| Aortic valve anomalies | 0/38 | 0.0 | 4/131 | 3.1 | 0.576 |
| Pulmonary valve anomalies | 1/38 | 2.6 | 6/131 | 4.6 | 1.000 |
| Mitral valve anomalies | 2/38 | 5.3 | 5/131 | 3.8 | 0.655 |
| Other cardiac anomalies | 5/38 | 13.2 | 12/131 | 9.2 | 0.540 |
| Respiratory malformations | 2/49 | 4.1 | 9/173 | 5.2 | 1.000 |
| Kidney malformations | 8/49 | 16.3 | 17/173 | 9.8 | 0.204 |
| Gastroenteric malformations | 5/49 | 10.2 | 20/173 | 11.6 | 1.000 |
| Genital malformations | 6/49 | 12.2 | 12/173 | 6.9 | 0.241 |
| Cryptorchidism | 4/49 | 8.2 | 7/173 | 4.0 | 0.265 |
| Hypospadias | 0/49 | 0.0 | 2/173 | 1.2 | 1.000 |
| Other genital anomalies | 3/49 | 6.1 | 4/173 | 2.3 | 0.182 |
| CNS malformations | 17/50 | 34.0 | 70/173 | 40.5 | 0.409 |
| Corpus callosum anomalies | 3/19 | 15.8 | 19/70 | 27.1 | 0.382 |
| White matter anomalies | 2/19 | 10.5 | 10/70 | 14.3 | 1.000 |
| Hippocampus anomalies | 3/19 | 15.8 | 10/70 | 14.3 | 1.000 |
| **Other CNS anomalies** | 19/19 | 100.0 | 56/70 | 80.0 | **0.035** |
| Epilepsy | 11/48 | 22.9 | 27/171 | 15.8 | 0.249 |
| **EEG anomalies** | 24/48 | 50.0 | 54/171 | 31.6 | **0.019** |
| Neurological anomalies | 15/49 | 30.6 | 72/173 | 31.6 | 0.164 |
| Dyspraxia | 2/49 | 4.1 | 9/173 | 5.2 | 1.000 |
| Hypotonia | 3/49 | 6.1 | 30/173 | 17.3 | 0.051 |
| Clumsiness | 5/49 | 10.2 | 13/173 | 7.5 | 0.557 |
| Other neurological anomalies | 11/49 | 22.4 | 40/173 | 23.1 | 0.921 |
| Hearing loss | 5/42 | 11.9 | 17/131 | 13.0 | 0.856 |
| Sensorineural hearing loss | 2/42 | 4.8 | 3/131 | 2.3 | 0.596 |
| Conductive hearing loss | 3/42 | 7.1 | 10/131 | 7.6 | 1.000 |
| Other hearing anomalies | 0/42 | 0.0 | 7/132 | 5.3 | 0.198 |
| Ocular anomalies | 14/42 | 33.3 | 58/128 | 45.3 | 0.173 |
| Astigmatism | 3/42 | 7.1 | 13/128 | 10.2 | 0.763 |
| Myopia | 2/42 | 4.8 | 8/128 | 6.3 | 1.000 |
| Hypermetropia | 2/42 | 4.8 | 10/128 | 7.8 | 0.731 |
| Strabismus | 9/42 | 21.4 | 33/128 | 25.8 | 0.570 |
| Exophoria | 2/42 | 4.8 | 4/128 | 3.1 | 0.638 |
| Exotropia | 3/42 | 7.1 | 5/128 | 3.9 | 0.410 |
| Esotropia | 3/42 | 7.1 | 14/128 | 10.9 | 0.596 |
| Other ocular anomalies | 2/42 | 4.8 | 17/128 | 13.3 | 0.164 |
| Dysmorphisms | 22/50 | 44.0 | 78/173 | 45.1 | 0.892 |
| Skull/face | 16/50 | 32.0 | 69/173 | 39.9 | 0.312 |
| Forehead/eyebrows | 13/50 | 26.0 | 54/173 | 31.2 | 0.479 |
| Eyes/eyelids/eyelashes | 21/50 | 42.0 | 61/173 | 35.3 | 0.474 |
| Hypertelorism | 4/50 | 8.0 | 8/173 | 4.6 | 0.474 |
| Epicanthus | 6/50 | 12.0 | 19/173 | 11.0 | 0.841 |
| Up-slanting palpebral fissures | 7/50 | 14.0 | 14/173 | 8.1 | 0.269 |
| Down-slanting palpebral fissures | 1/50 | 2.0 | 13/173 | 7.5 | 0.201 |
| Other eye dysmorphisms | 10/50 | 20.0 | 28/173 | 16.2 | 0.527 |
| Nose | 13/50 | 26.0 | 54/173 | 31.2 | 0.479 |
| Philtrum | 4/50 | 8.0 | 25/173 | 14.5 | 0.232 |
| Mouth/teeth/tongue | 17/50 | 34.0 | 76/173 | 43.9 | 0.210 |
| Ears | 27/50 | 54.0 | 74/173 | 42.8 | 0.160 |
| Neck/chest | 6/50 | 12.0 | 17/173 | 9.8 | 0.656 |
| Limbs | 21/50 | 42.0 | 90/173 | 52.0 | 0.212 |
| **Hands** | 10/50 | 20.0 | 60/173 | 34.7 | **0.049** |
| Hand brachydactyly | 3/50 | 6.0 | 8/173 | 4.6 | 0.731 |
| Hand clinodactyly | 3/50 | 6.0 | 23/173 | 13.3 | 0.157 |
| Hand syndactyly | 0/50 | 0.0 | 1/173 | 0.6 | 1.000 |
| Hand camptodactyly | 0/50 | 0.0 | 7/173 | 4.0 | 0.354 |
| Arachnodactyly | 3/50 | 6.0 | 9/173 | 5.2 | 0.734 |
| Other hand dysmorphisms | 3/50 | 6.0 | 29/173 | 16.8 | 0.056 |
| Flat feet | 6/50 | 12.0 | 14/173 | 8.1 | 0.404 |
| Foot syndactyly | 2/50 | 4.0 | 8/173 | 4.6 | 1.000 |
| Other foot dysmorphisms | 11/50 | 22.0 | 41/173 | 23.7 | 0.802 |
| **Lower limb dysmorphisms** | 1/50 | 2.0 | 27/172 | 15.7 | **0.010** |
| Scoliosis | 2/50 | 4.0 | 19/173 | 11.0 | 0.175 |
| Other skeletal dysmorphisms | 5/50 | 10.0 | 30/173 | 17.3 | 0.209 |
| Hair dysmorphisms | 3/50 | 6.0 | 18/173 | 10.4 | 0.423 |
| Nail dysmoprhisms | 2/50 | 4.0 | 11/173 | 6.4 | 0.738 |
| Skin anomalies | 10/50 | 20.0 | 46/173 | 26.6 | 0.344 |
| Delayed bone age | 2/50 | 4.0 | 6/173 | 3.5 | 1.000 |
| Advanced bone age | 3/50 | 6.0 | 23/173 | 13.3 | 0.213 |
| Hypothyroidism | 0/50 | 0.0 | 2/173 | 1.2 | 1.000 |
| Obesity | 0/50 | 0.0 | 3/173 | 1.7 | 1.000 |
| GH deficiency | 1/50 | 2.0 | 8/173 | 4.6 | 0.688 |
| Other endocrinolagical anomalies | 0/50 | 0.0 | 4/173 | 2.3 | 0.557 |
| Skin softness | 0/50 | 0.0 | 1/173 | 0.6 | 1.000 |
| Joint laxity | 2/50 | 4.0 | 7/173 | 4.0 | 1.000 |

Statistically significant results for likely pathogenic VUS are reported in bold [n/N, number of cases with positive variable/number of patients with available data on that variable; NA: not applicable; ADHD: Attention deficit and hyperactivity disorder; ASD: atrial septal defect; CNS: central nervous system; CTG: fetal cardiotocography; IUGR: intrauterine growth restriction; PDA: patent ductus arteriosus; PFO: patent foramen ovale; ToF: Tetralogy of Fallot; VSD: interventricular septal defect; VUS: variants of uncertain significance]
