# Supplementary material for: Prematurity, ventricular septal defect and dysmorphisms are independent predictors of pathogenic copy number variants: a retrospective study on array-CGH results and phenotypical features of 293 children with neurodevelopmental disorders and/or multiple congenital anomalies
Source: Ital J Pediatr. 2018 Mar 9;44:34. doi: 10.1186/s13052-018-0467-z (PMC5845186; doi:10.1186/s13052-018-0467-z)
Supplement: Supplementary file 8 — Table S8. Correlations between phenotypical core features and aCGH results (positive aCGH vs negative aCGH). Statistically significant results for negative aCGH are reported in bold; statistically significant results for positive aCGH are reported in bold and italic. [MCA: multiple congenital anomalies; NDD: neurodevelopmental disorders]. (DOC 49 kb) [file 13052_2018_467_MOESM8_ESM.doc]

|  | positive aCGH  (N = 225) | | negative aCGH  (N = 68) | | p value |
| --- | --- | --- | --- | --- | --- |
|  | n | % | n | % |  |
| **NDD** | 52 | 23.11 | 31 | 45.59 | **0.000312** |
| Dysmorphism(s) | 2 | 0.89 | 0 | 0 | NA |
| MCA | 14 | 6.22 | 8 | 11.76 | 0.12856 |
| Epilepsy | 3 | 1.33 | 0 | 0 | NA |
| ***NDD+Dysmorphism(s)*** | 53 | 23.56 | 8 | 11.76 | ***0.035858*** |
| NDD+MCA | 30 | 13.33 | 8 | 11.76 | 0.73582 |
| NDD+epilepsy | 14 | 6.22 | 3 | 4.41 | 0.575742 |
| NDD+dysmorphism(s)+MCA | 26 | 11.56 | 4 | 5.88 | 0.176283 |
| NDD+dysmorphism(s)+epilepsy | 4 | 1.78 | 0 | 0.00 | NA |
| NDD+MCA+epilepsy | 9 | 4.00 | 2 | 2.94 | 0.687305 |
| NDD+dysmorphism(s)+MCA+epilespy | 2 | 0.89 | 0 | 0 | NA |
| Dysmorphism(s)+MCA | 11 | 4.89 | 3 | 4.41 | 0.871591 |
| Dysmorphism(s)+epilepsy | 0 | 0 | 0 | 0 | NA |
| MCA+epilepsy | 4 | 1.78 | 1 | 1.47 | 0.863911 |
| Dysmorphism(s)+MCA+epilepsy | 0 | 0 | 0 | 0 | NA |
| Other | 1 | 0.44 | 0 | 0 | NA |

**Table S8 - Correlations between phenotypical core features and aCGH results (positive aCGH vs negative aCGH).**

**Correlations between phenotypical core features and aCGH results (positive aCGH vs negative aCGH). Statistically significant results for negative aCGH are reported in bold; statistically significant for positive aCGH are reported in bold and italics. [MCA: multiple congenital anomalies; NDD: neurodevelopmental disorders]**
